# EGFR-Targeted Photodynamic Therapy

**DOI:** 10.3390/pharmaceutics14020241

**Published:** 2022-01-20

**Authors:** Luca Ulfo, Paolo Emidio Costantini, Matteo Di Giosia, Alberto Danielli, Matteo Calvaresi

**Affiliations:** 1 Dipartimento di Farmacia e Biotecnologie, Alma Mater Studiorum—Università di Bologna, Via Francesco Selmi 3, 40126 Bologna, Italy; luca.ulfo2@unibo.it (L.U.); paolo.costantini4@unibo.it (P.E.C.); 2 Dipartimento di Chimica “Giacomo Ciamician”, Alma Mater Studiorum—Università di Bologna, Via Francesco Selmi 2, 40126 Bologna, Italy; matteo.digiosia2@unibo.it

**Keywords:** EGFR, PDT, targeting, antibodies, EGF, ligands, nanobodies, affibodies, aptamers, phages

## Abstract

The epidermal growth factor receptor (EGFR) plays a pivotal role in the proliferation and metastatization of cancer cells. Aberrancies in the expression and activation of EGFR are hallmarks of many human malignancies. As such, EGFR-targeted therapies hold significant potential for the cure of cancers. In recent years, photodynamic therapy (PDT) has gained increased interest as a non-invasive cancer treatment. In PDT, a photosensitizer is excited by light to produce reactive oxygen species, resulting in local cytotoxicity. One of the critical aspects of PDT is to selectively transport enough photosensitizers to the tumors environment. Accordingly, an increasing number of strategies have been devised to foster EGFR-targeted PDT. Herein, we review the recent nanobiotechnological advancements that combine the promise of PDT with EGFR-targeted molecular cancer therapy. We recapitulate the chemistry of the sensitizers and their modes of action in PDT, and summarize the advantages and pitfalls of different targeting moieties, highlighting future perspectives for EGFR-targeted photodynamic treatment of cancer.

## 1. Anticancer Photodynamic Therapy

### 1.1. Photodynamic Therapy: An Overview

Photodynamic therapy (PDT) is a clinically approved, minimally invasive procedure for cancer treatments. The combination of a photosensitizer (PS), light of appropriate wavelength, and in situ molecular oxygen (O_2_) produces local photodamage, triggering a series of cell death mechanisms.

PDT is a two-step procedure, starting with the administration of a PS agent, which should accumulate preferentially in cancer tissues. After a defined time (drug–light interval), the sensitizer is activated by a light source, whose wavelength matches its absorbance band. Due to the presence of oxygen, a cascade of events occurs, resulting in direct tumor cell death, microvascular damage, and initiation of local inflammatory responses [1,2].

PDT offers many advantages compared to conventional treatment methods, including minimal invasiveness, repeatability without cumulative toxicity, spatial and temporal control, excellent functional and cosmetic results, reduced long-term morbidity, and improved quality of life of patients. If chemotherapeutic drugs induce systemic toxicity and ionizing radiation of radiotherapy damages neighboring healthy tissues, each component employed by PDT does not generally have toxic effects *per se* on biological systems [2,3]. The main advantage of PDT is the possibility to focus the irradiation locally at the desired site of action, lowering the collateral damage to healthy tissues. PDT can be used in combination with chemotherapy or radiotherapy, without compromising these therapeutic modalities, or as an adjunctive treatment following surgical resection of the tumor to reduce residual tumor burden [2].

Despite the advantages of PDT, its clinical application in cancer therapy is limited to superficial and endoscope- or surgery-accessible regions. This is mainly due to the limited tissue penetration depth of light. When visible light radiation interacts with tissues, reflection, refraction, scattering, and absorption phenomena contribute to the overall reduction in light intensity. As the tissue thickens, the rapid depletion of the light dose causes an ineffective treatment [1,4].

Lower absorption and reduced scattering phenomena can be obtained using near-infrared (NIR) radiation. In fact, the region between 600 and 1300 nm is known as the “optical window” of biological tissue, which allows a deeper penetration of light (>6 mm). The most common therapeutic window used for PDT applications is between 600 and 800 nm [4,5].

With the development of multi-photon lasers, two-photon excitation was investigated for PDT. The absorption of two photons of light offers two advantages: (i) it allows spatially precise activation of photosensitizers in tissues; (ii) it produces the same excited state that would have been produced by one-photon excitation after absorbing twice the energy [6,7]. Valuable alternatives are molecular antennae, acting as energy donor species toward the PS [8,9,10,11] and upconverting nanoparticles [12].

### 1.2. Photophysical and Photochemical Mechanisms of PDT

**Figure 1:**
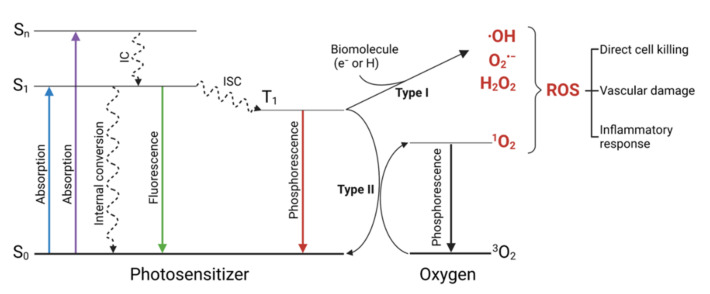
Jablonski diagram of photosensitizer (PS) excited states showing the photochemical mechanisms operating in photodynamic anticancer therapy.

When irradiated with the appropriate wavelength, a PS absorbs one photon and is promoted from its ground state (S_0_) to the first singlet excited state (S_1_) or to higher singlet excited states (S_n_). S_n_ rapidly decay (~fs) to S_1_ through internal conversion (IC). The PS in the S_1_ excited state is unstable, with a lifetime in the range of ns, resulting in decay to the ground state S_0_ through a (i) radiative (fluorescence) or (ii) non-radiative (energy dissipation as heat) relaxation process (Figure 1).

A third pathway may occur when the singlet−triplet energy gap is sufficiently small: an intersystem crossing (ISC) from S_1_ to T_1_ [13,14]. The T_1_ excited state is generally characterized by a long lifetime (from μs to s) and can be subjected to different photophysical and photochemical processes, such as (i) phosphorescent emission and (ii) generation of reactive oxygen species (ROS). Reactive oxygen species may be generated through two alternative pathways: an electron-transfer mechanism (type I) or an energy transfer process (type II) [7,15,16]. In the type I mechanism, T_1_ reacts directly with a biomolecule in a cellular microenvironment, acquiring a hydrogen atom or an electron to form a radical, which further reacts with H_2_O or molecular oxygen (^3^O_2_), leading to the production of different radical oxygen species, such as superoxide anion (O_2_^•−^), hydroxyl (^•^OH) radicals, and hydrogen peroxide (H_2_O_2_). In the type II mechanism, an energy transfer between the T_1_ state of PS to ^3^O_2_ occurs, forming a highly reactive singlet oxygen excited state (^1^O_2_) [17,18]. Type I and type II processes are not independent but instead can influence and even promote each other. The two types of photodynamic reactions can occur simultaneously, and the contribution of each of the two processes is affected by several factors related both to the biological environment (substrates, medium, local polarity, oxygen concentration) and physicochemical properties of the PS. The principal targets of ROS, subjected to irreversible degradation, are electron-rich biomolecules, such as aromatic amino acids and unsaturated lipids. ^•^OH is the most toxic ROS because it may attack the majority of organic biomolecules, including lipids, carbohydrates, proteins, amino acids, nucleic acids, and DNA [19,20,21]. Additionally, ^1^O_2_ can damage biotissues irreversibly, resulting in the degradation and oxidation of the membrane. In contrast, O_2_^•−^ is not a strong oxidant; however, it contributes to the alteration of the ROS homeostasis and stress signaling pathways and is a precursor of ^•^OH and ^1^O_2_ [22]. Ultimately, the oxidative stress in physiological processes is mainly attributed to ^•^OH and ^1^O_2_ [15].

### 1.3. Mechanisms for Photodynamic-Therapy-Induced Cancer Cell Death

The efficacy of PDT-mediated tumor killing depends on several intercorrelated variables, such as the type, concentration, and cellular localization of the PS; the type and oxygenation level of the tumor; as well as the light fluence rate and total fluence [4,23,24]. Once properly activated, PSs induce tumor damage through three cooperative pathways: (i) direct cytotoxicity on tumor cells; (ii) tumoral vascular system impairment; (iii) stimulation of inflammatory reactions.

#### 1.3.1. Direct Cytotoxicity on Tumor Cells

ROS generated from photoinduced PSs interact with and alter a wide range of molecules (proteins, lipids, nucleic acids, amino acids), causing irreversible photodamage in different cellular compartments. Considering the short lifetime of ROS (10 to 320 ns for ^1^O_2_), their diffusion range in cells is restricted to 10–55 nm [25]. Thus, the cellular localization of activated PS determines the subcellular area to be photodamaged, severely impacting the fate of cells. In general, the PDT-mediated killing of cancer cells occurs through three main mechanisms: apoptosis, necrosis, and autophagy [1]. Photodamage at the level of the mitochondria outer membrane leads to its permeabilization, resulting in the activation of proapoptotic proteins, such as AIF (apoptotic-inducing factor) or caspase activators (Smac/DIABLO, cytochrome c), which trigger programmed cell death [1,26]. Furthermore, apoptosis induced via mitochondrial outer membrane permeabilization can occur when proteases (cathepsins) are released in the cytosol via the photodynamic disruption of lysosomes [27]. Alternatively, excessive cellular damage or the blocking of apoptotic pathways shifts the cell’s fate from apoptotic cell death to necrosis. Necrosis is typically characterized by the disruption of organelles, the nucleus, and cell membranes, with the consequent spillage of cell contents in the extracellular environment, followed by the activation of a strong inflammatory response and tissue damage [28]. Autophagy, also known as macroautophagy, is a controlled lysosomal pathway involved in the recycling of damaged proteins or organelles that is stimulated by several stressors, including PDT-mediated oxidative stress. Autophagy stimulation can lead to cell death, although it was also demonstrated to be involved in cancer cells resistance to PDT treatment by providing protection from photodamage and recycling of impaired organelles [1,28,29].

#### 1.3.2. Tumoral Vascular System Impairment

Vascularization is crucial for solid tumor growth, allowing nutrient delivery to cancer cells, and is usually stimulated by the tumor itself. The rapid angiogenesis and incomplete cellular borders provoke the formation of both blood and lymphatic leaky vessels, which help the delivery and accumulation of PSs to target cells [2]. It was proven that tumoral vascularization is critically injured after PDT treatment, with increased hypoxia levels and reduced tumor growth. Indeed, the photoactivation of PSs localized on or in the proximity of endothelial cells promote the detachment of endothelial tissues from the vascular basement membrane. This process deeply impacts the vasculature stability by producing thrombogenic regions characterized by thrombocyte aggregation, the production of vasoactive molecules, and increased vessel permeability and constriction [2,30].

#### 1.3.3. Immunostimulatory Effect

The stimulation of both innate and adaptive immunity strongly enhances PDT activity by producing a secondary cytotoxic effect on cancer cells and providing long-term tumor protection. The localized oxidative stress generated by PDT, together with its cytotoxic effect on cancer cells and the vascular damage, induces an acute inflammatory response. Neutrophils, attracted by the signaling molecules DAMPs (damage-associated molecular patterns) and CDAMPs (cell death-associated molecular patterns), rapidly invade tumors and recruit mast cells and macrophages [2,31]. Macrophages and dendritic cells present tumor-derived antigens to CD4 helper lymphocytes, which in turn activate CD8 T lymphocytes that can then induce apoptosis or necrosis in other cancer cells [32,33]. The resulting inflammatory process was demonstrated to also be regulated by cytokines. IL-1β and IL-6, in particular, seem to be crucial for PDT [34]. The immunostimulatory effect of PDT can vary depending on (i) the type of PS used, (ii) the body site, and (iii) the type of tumor [1,35]. In this regard, while the majority of PDT treatments result in an immunostimulatory effect, epidermal and transepidermal PDT treatments of large surfaces are associated with immunosuppression [36].

### 1.4. Photosensitizers

**Figure 2:**
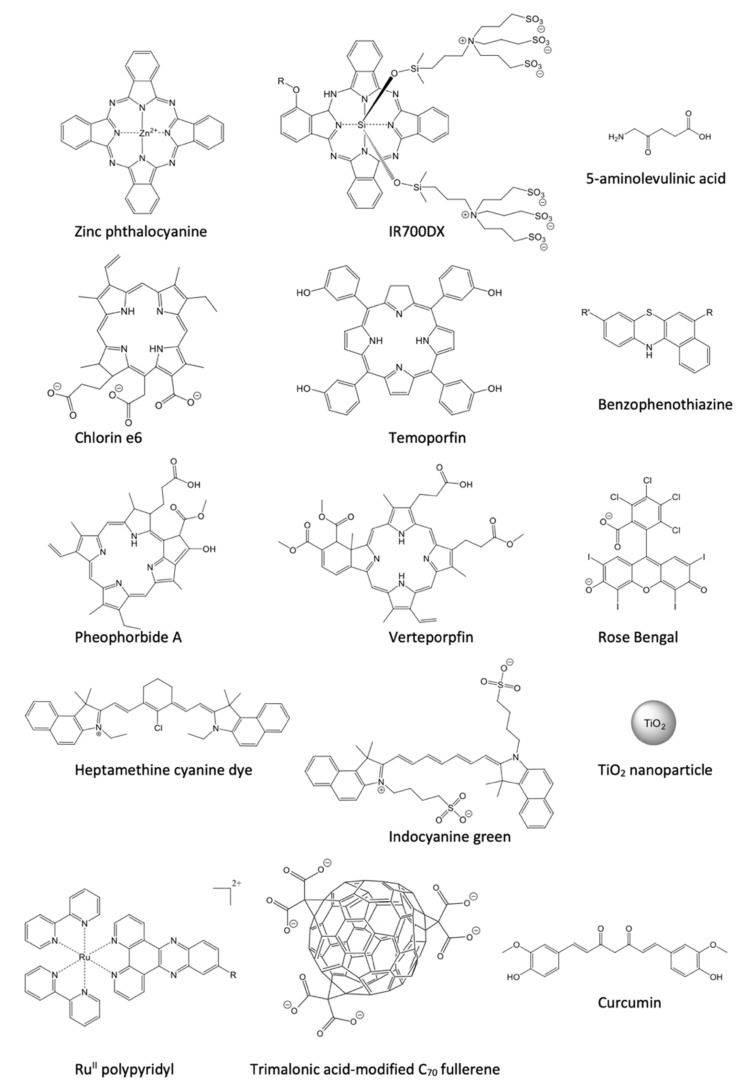
Representative photosensitizers (PS) used in EGFR-targeted PDT.

To find application in photodynamic cancer therapy, a photosensitizer (Figure 2) should meet specific criteria. Firstly, it must accumulate preferentially in cancerous tissues and rapidly be cleared from the healthy ones. The singlet excited state must undergo intersystem crossing with a high quantum yield to form a long-lived triplet excited state, allowing an efficient interaction with molecular oxygen and biomolecules. Based on the biological environment, a PS should be able to produce ROS through both the mechanisms (Figure 1). To ensure the selectivity of the treatment, dark toxicity must not occur. A high absorption coefficient above 700 nm is preferred to activate the PS accumulated in deep tissues [2,5,17]. The design of new photosensitizing molecules must consider that a clinically successful PS must be amphiphilic. In fact, a certain degree of hydrophilicity is required to prevent aggregation and travel toward the target tissue, where the lipophilic component promotes the diffusion across the plasma membrane [2]. Most photosensitizers localize outside the nucleus, minimizing the genotoxic and mutagenic potential of PDT [37].

#### 1.4.1. First-Generation PSs

The first-generation photosensitizers were hematoporphyrin derivatives (HpD), which were developed in the 1970s and early 1980s. Photofrin^®^ is a mixture of HpD (monomers, dimers, and oligomers). It was the first PS clinically approved (in 1993, for bladder cancer) and one the most used PS for cancer treatments [38]. HpD suffer from relatively low absorption of light in the spectral transparency window, where the light penetration of tissues is optimal for PDT. Due to their low absorption coefficients, the administration of a high dose of PS is necessary to achieve a sufficient phototherapeutic response [17]. In addition, the low preferential accumulation in cancer tissues, the scarce bioavailability, the poor pharmacokinetics, and the risks of lingering photosensitivity in healthy tissues (e.g., eyes and skin) for weeks hampered the applications of first-generation photosensitizers during their initial clinical trials.

#### 1.4.2. Second-Generation PSs

To overcome these limitations, second-generation PSs were developed [16]. Most of them are porphyrinoid compounds such as chlorins (i.e., temoporfin, Foscan^®^) [39], bacteriochlorins, phthalocyanines (e.g., Photosens) [40], pheophorbides, bacteriopheophorbies, and texaphyrins. Additionally, non-porphyrinoid compounds such as anthraquinones, phenothiazines, xanthenes, cyanines [41], fullerenes [8,42], borondipyrromethene [43], and curcuminoids [44] have attracted wide clinical interest. Their main distinguishing feature lies in the longer absorption wavelengths (>600 nm), characterized by a high extinction coefficient (>5 × 10^4^ M^−1^ cm^−1^).

Compared to first-generation PSs, several features such as the high quantum yields of ROS production and preferential accumulation to tumoral cells have been enhanced, exhibiting overall better antitumor effect. The shorter accumulation times in cancer cells and faster clearance from normal tissues allow reductions in the drug–light interval and post-treatment cutaneous photosensitivity, making second-generation PSs more suitable for clinical applications.

#### 1.4.3. Third-Generation PSs

The third generation of PSs appeared as an attempt to achieve compounds with improved delivery and tumor targeting, conjugating first- or second-generation PSs with targeting agents. A careful selection of the linker geometry is necessary to maintain both the recognition ability of the targeting moiety and the photodynamic performance of the free PS. Special care should be taken in choosing the targeting moiety, linker, and PS, because the PDT treatment can trigger the relocalization of the PS to another subcellular domain or the extracellular media. This relocalization may be a consequence of the degradation (i) of the light-absorbing photosensitizer itself, (ii) of the biomolecular carrier or chemical linker, or (iii) of the biomolecules in the environment surrounding the PS (i.e., lipids or proteins) [45] due to the generated oxidative stress.

Although the light activation of the photosensitizer allows for a certain degree of local selectivity and the excitation light itself is harmless, conventional PDT is still limited by certain drawbacks:

(1) Phototoxicity and photosensitivity in healthy tissues. Most PSs are poorly selective molecules that bind not only cancer cells but also normal cells, including the skin and other epithelial tissues, resulting in unwanted phototoxicity and photosensitivity (i.e., eyes and skin). Of course, if compared to chemotherapeutics, the local activation by light, needed to exert photodynamic activity, reduces the likelihood of off-target effects;
(2) Poor biodistribution. PSs have the same limitations as cancer chemotherapeutic drugs in terms of delivery; that is, direct parenteral administration through intravenous injection leads to unpredictable biodistribution. Because of the non-specific biodistribution, considerable drug losses and inadequate PS concentrations at the target may occur;
(3) Hydrophobicity and the need for formulants. Many PSs are highly hydrophobic. Accordingly, they need to be administrated through intravenous formulations such as cremophor, ethanol, or propylene-glycol-based excipients. This determines a poorly controlled (re)distribution of the PS molecules towards plasma proteins and other off-target tissues, as well as hypersensitivity and toxicity caused by the excipients, especially if repeated treatments are required.

#### 1.4.4. Receptor-Targeted PSs

**Figure 3:**
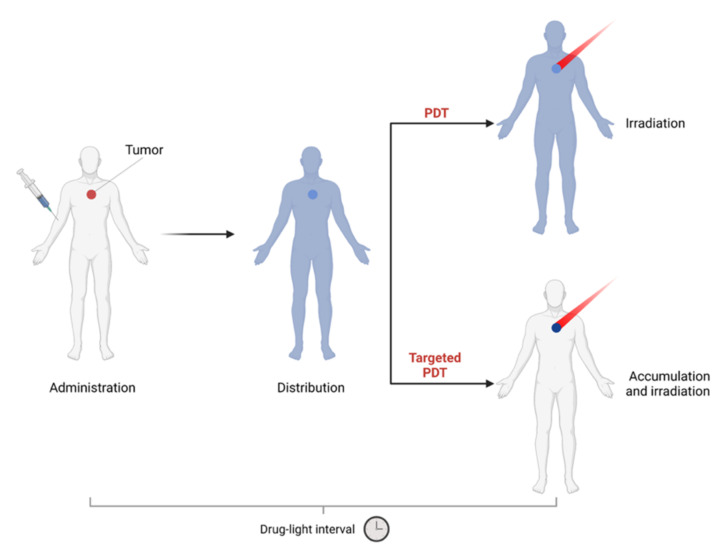
Therapeutic application of PDT or targeted PDT. The patient is administered with the photosensitizer, which concentrates at the tumor. The photosensitizer is then activated by light, destroying the tumor. Created with BioRender.com.

In order to overcome these issues, photosensitizers can be conjugated to targeting moieties that specifically bind to receptors overexpressed on tumor cells (active targeting), leading to improved internalization at the site of interest and decreasing unwanted off-target effects (Figure 3).

Receptor-targeted PSs administered systemically have a higher affinity for cancer cells and a low affinity for healthy tissue, improving the therapeutic efficiency of PDT. This is clinically important, since it reduces the systemic toxicity without affecting the higher therapeutic dosages needed to locally activate PDT. In addition, the conjugation with targeting biomolecules also improves the solubility of the PS, eliminating the need for formulants.

## 2. Epidermal Growth Factor Receptor (EGFR)

Surface receptors and endogenous signaling molecules, specifically expressed or activated in tumors, represent excellent targets for the development of effective cancer therapies, since targeted treatments minimize the risk of side-effects that often accompany untargeted therapies [46].

Among the various oncotargets identified, the epidermal growth factor receptor (EGFR) family of tyrosine kinase receptors represents a particularly appealing one, due to the amplification, overexpression, and gain of function of EGFRs in a wide plethora of tumors [47,48].

### 2.1. EGFR Biology

**Figure 4:**
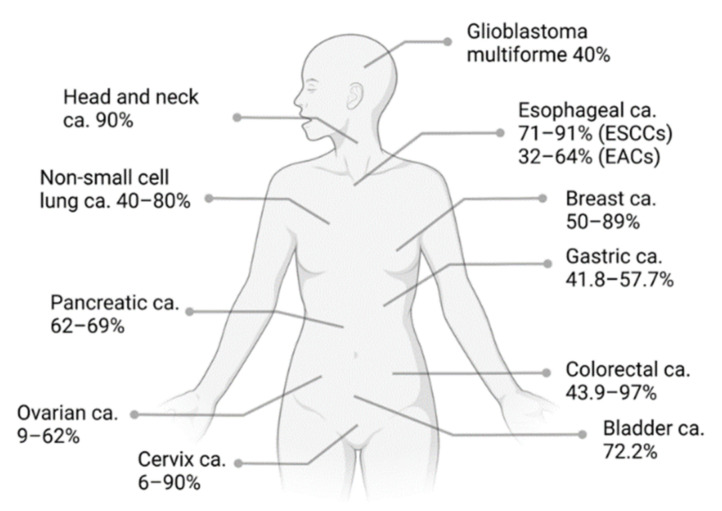
The positivity proportions of EGFR expression in various cancers (ca., carcinoma; ESCC, esophageal squamous cell carcinoma; EAC, esophageal adenocarcinoma). Adapted from Kato et al., Cancers, published by MDPI in 2021. Created with BioRender.com.

EGFR, also known as ERBB1 or HER1, is a transmembrane tyrosine kinase receptor involved in crucial aspects of epithelial cell physiology, such as growth, differentiation, and motility [49]. It belongs to the ErbB family of receptor tyrosine kinases (RTKs), together with HER2 (also known as ERBB2), HER3 (also known as ERBB3), and HER4 (also known as ERBB4) [50]. EGFR is encoded by the *EGFR* gene, located in the p-arm of chromosome 7. The mature product is a 170 kDa transmembrane glycoprotein composed of 1186 aa [51]. Increased levels of *EGFR* expression or increased gain-of-function due to gene amplification, duplication, mutations, deletions, or in-frame alteration is frequent in several human cancers and is associated with an adverse prognosis [52,53]. Indeed, the overexpression of EGFR was detected in colorectal, lung, breast, ovarian, cervical, bladder, esophageal, stomach, brain, neck, and endometrial cancers [50,53,54,55] (Figure 4).

**Figure 5:**
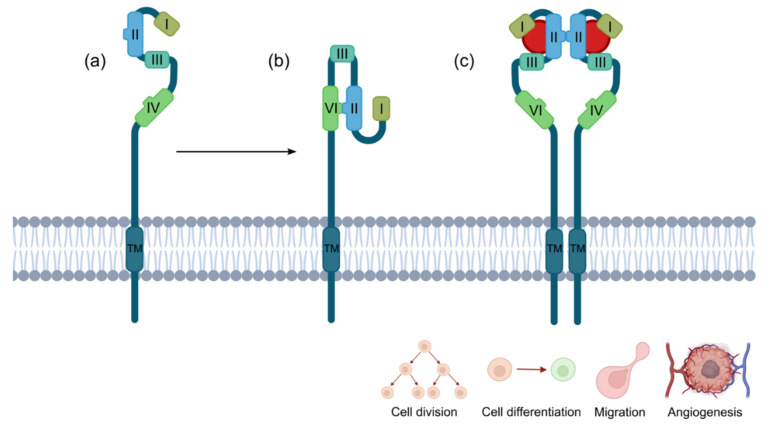
EGFR structure and conformation: (**a**) open conformation (active) and (**b**) closed conformation (inactive); (**c**) the ligand binding drives EGFR dimerization and activates the signaling cascade, with consequent stimulation of cell division and differentiation, as well as migration and angiogenesis. Created with BioRender.com.

Domain-wise, EGFR is constituted by a ligand binding extracellular region (N-terminal), a transmembrane portion containing an alpha helix peptide, and an intracellular kinase region (C-terminal) (Figure 5).

The extracellular domain (residues 25–641) is composed of four subdomains (I-IV) and can be in an open (also known as active) or closed (inactive) conformation. Subdomains I and III present similar topologies and are involved in the binding to EGF, while II and IV are cysteine-rich regions implicated in the receptor dimerization [56,57,58]. In the inactive conformation, the intermolecular interaction between the II and IV subdomains prevents ligand binding, while in the open conformation EGFR presents a C-shape with I and III accessible for EGF [59,60]. The binding of EGF to subdomains I and III induces a structural rearrangement, which allows the dimerization domain, encompassed in II, to interact with the subdomain II of another active EGFR, thereby driving homodimerization. The crystal structure resolution of the EGF–EGFR complex demonstrated that the side chains of Leu14, Tyr45, Leu69, and Leu98 of subdomain I establish hydrophobic interactions with Met21, Ile23, and Leu26 of EGF [56]. In subdomain III, side chains of Leu382, Phe412, Val350, Phe357, and Ile438 hydrophobically interact with Leu15, Tyr13, and Leu47 of EGF, while Asp355 forms a salt bridge with Arg41 of the growth factor [56]. The intracellular region of EGFR (residues 669–1210) is composed of a membrane proximal juxtamembrane segment (669–705), a tyrosine kinase domain (706–979), and a C-terminal tail of 229 amino acids [58,61]. Receptor homodimerization in the extracellular compartment brings the intracellular domains in proximity and allow the formation of an asymmetric kinase domain dimer [61]. In particular, the activation of kinase domain is due to allosteric interactions between the C-lobe (activator) of the kinase domain present in one receptor and the N-lobe (receiver) displayed by the second receptor of the homodimer. This interaction activates the receiver kinase that *trans*-phosphorylate tyrosine residues in the C-terminal tail of the ‘activator’ kinase present in the first receptor [62,63,64,65]. The phosphorylated tyrosine at the C-tail of EGFR recruits and activates several signaling proteins that initiate the signal transduction cascade, eventually triggering numerous pathways involved in cell proliferation, survival, apoptosis, migration, angiogenesis, and differentiation [49,66,67].

**Figure 6:**
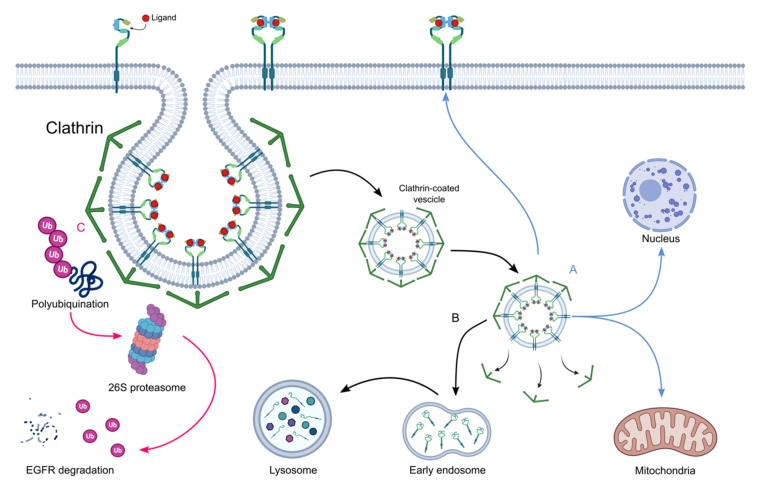
EGFR internalization, degradation, and reuse pathways. The active homodimer of EGFR is internalized through clathrin-coated vesicles, meaning (**A**) ligand-free receptors can be recycled in cell membrane. Alternatively, active EGFR may escape the degradation process and become tagged to the plasma membrane, nucleus, and mitochondria (blue arrows). (**B**) EGFR–ligand complexes are routed to lysosomes for degradation (black arrows) or (**C**) degraded via the proteosome pathway (pink arrows). Created with BioRender.com.

EGFR activity is controlled by three main mechanisms that mitigate the signal by removing the tyrosine kinase receptor from the cell membrane: the clathrin pathway, the proteasome pathway, and clustering-induced internalization (Figure 6).

In clathrin-mediated endocytosis, which is the main EGFR internalization pathway, EGFR is internalized by the formation of clathrin-coated intracellular vesicles that are then fused with early endosomes. At this step, ligand-free receptors can be recycled to cell surfaces while EGFR–ligand complexes are routed to late endosomes and then to lysosomes for degradation [68,69,70]. In the proteasome pathway the E3 ligase Cbl, in complex with the adaptor protein Grb2, recognizes the phosphorylated tyrosine at the C-tail of EGFR and induces polyubiquitination of EGFR receptor, which is further degraded via the 26 s proteasome [71,72]. Alternatively, EGFR can be internalized via a clustering-induced mechanism that is mediated by the dimerization motif in the transmembrane domain [73]. It was also demonstrated that activated EGFR can escape degradation and be recycled in the plasma membrane or tagged to cellular organelles, such as mitochondria (either on the inner and outer membrane) and nuclei [74,75,76,77]. Once translocated within these organelles, EGFR acquires novel functions and can be involved in transcriptional regulation by acting as a co-transcriptional factor, DNA double-strand break repair, mitochondrial dynamics, and ATP production [78,79,80,81,82]. In general, nuclear and mitochondrial EGFR localization is associated with severe prognosis in patients with lung, ovarian, breast, and oropharyngeal cancers [78,83,84].

### 2.2. EGFR-Targeted Cancer Therapies

Disruption or downregulation of aberrant EGFR activity has been shown to have potent antitumor effects. Monoclonal antibody treatment, readily followed by small-molecule inhibition of the receptor kinase signaling activity, was demonstrated to be an excellent targeting therapy, paving the road for the upsurge of many nanobiotechnological strategies, also involving peptides, aptamers, single-chain (scFv) and single-domain antibodies (sdAb, nanobodies), as well as other non-immunoglobulin folds (DARPins, Affibodies, etc.) as targeting moieties.

Significant inhibition of tumor cell growth and metastasis is also accomplished via the involvement of the immune system of cancer patients. These cancer immunotherapies counter the activity of immunosuppressive proteins expressed by cancer cells and trigger the cellular immune responses against the tumor cells [85]. In this respect, the synergistic application of nanoparticle delivery strategies to cancer immunotherapy represents a very promising approach, since this is believed to facilitate selective cytotoxicity by combining innovative ablation strategies with the humoral and cellular immune responses needed for cancer eradication [86].

A growing body of evidence demonstrates that unique weapons for selective ablation of cancer cells can be generated via the engineering of bona-fide EGFR-targeting moieties with light-activable PDT sensitizers or nanoparticles. These nanobiotechnological adducts provide a potent double-targeting therapy, exploiting the recognition ability of the targeting moieties and the possibility of focused irradiation, localized at the desired site of action, lowering the collateral damage to healthy tissues (Figure 3). As such, EGFR-targeted PDT approaches hold tremendous translational potential.

### 2.3. EGFR-Targeted PDT

**Figure 7:**
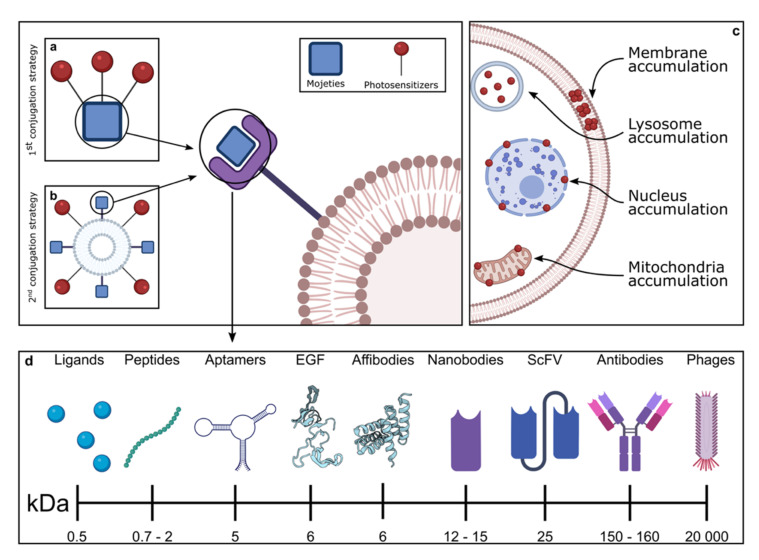
PDT conjugation strategies and targeting agents. (**a**) Direct conjugation of a PS to an EGFR-targeting agent. (**b**) Surface modification of a nanovector, incorporating a photosensitizer payload with EGFR-targeting agents. (**c**) Cellular localization of PSs after interaction between the targeting agent and EGFR receptor. (**d**) Different targeting agents used in EGFR-targeted PDT and their dimension in kDa. Created with BioRender.com.

Any of the validated targeting agents against EGFR may be exploited to selectively vehiculate the photosensitizers for PDT. In general, to obtain EGFR-targeting PSs, it is possible to use two alternative methods (Figure 7).

One such method is the conjugation of a first- or second-generation PS to biomolecules or ligands with EGFR-targeting ability (Figure 7a). The other method makes use of nanovectors modified with EGFR-targeting moieties (Figure 7b) as delivery vehicles for PSs.

For the former, the complicated structure and low stability of the biomolecules make the synthesis and purification of the PS conjugates problematic, and more importantly the recognition activities of the targeting agents are often changed or even lost during the conjugation procedures. For these reasons, a limited number of PSs may be conjugated to the targeting agent. For the latter, in addition to the synthetic problems described above, the procedures for surface modification with the targeting agents may strongly modify the size, charge, shape, stability, drug loading, and releasing ability of the nanovectors.

In principle, the higher the multivalency of the adducts cargoed to EGFR, the higher the expected production of cytotoxic ROS species upon irradiation.

The EGFR-mediated intracellular redirection of the sensitizers to specific organelles (Figure 7c) may contribute to eliciting different modes of cell death (i.e., apoptotic vs. necrotic) that could significantly impact the overall efficacy and tolerance of the treatment.

**Figure 8:**
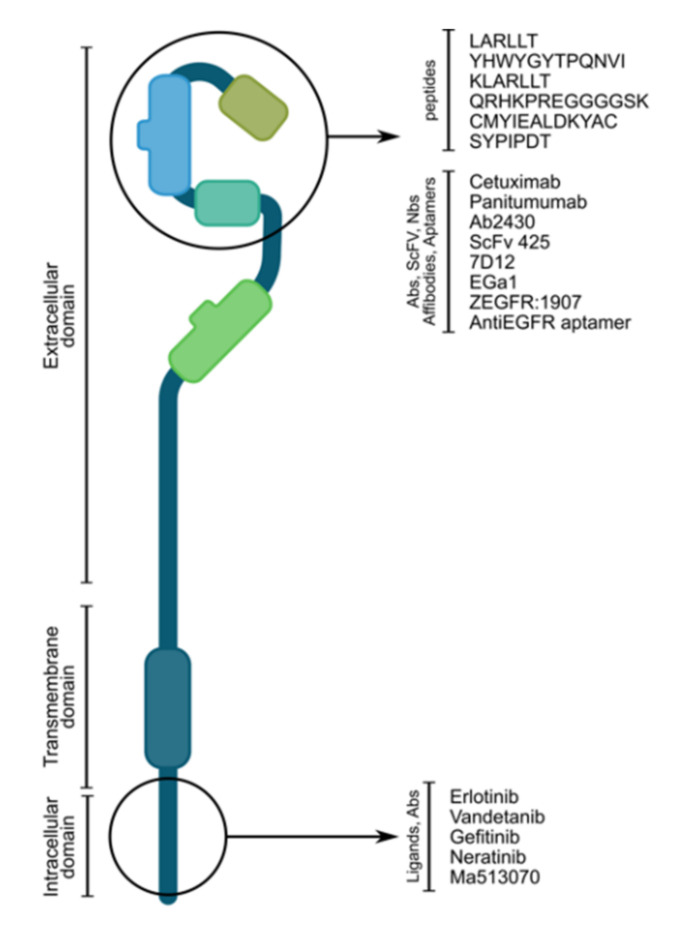
Domains of EGFR recognized by different targeting agents. Created with BioRender.com.

Importantly, the (bio)chemical nature and size of the targeting moiety (Figure 7) and the EGFR domain that it recognizes (Figure 8) may account for differences in tissue distribution and penetration, body clearance, cell internalization, achievable multivalency (number of sensitizers that can be delivered per binding event), and interplay with the immune system.

## 3. Targeting Agents Used in EGFR-Targeted Photodynamic Therapy

Below we systematically review the different targeting moieties that have been exploited for EGFR-targeted PDT, enumerating the different adducts with photosensitizers and their effects in vitro, in vivo, or ex vivo.

### 3.1. Epidermal Growth Factor (EGF)

EGF ligands drive proliferative signal transduction pathways through binding to the EGFR (Erb1) subclass of the receptor tyrosine kinase superfamily, influencing cellular proliferation, stem cell identity, and lineage differentiation. The EGF family of ligands includes eleven paralogues, sharing similar EGF-like motifs. When anchored to the membrane they act as juxtacrine signaling molecules between two neighboring cells. The proteolytic processing of the external domain promotes the release in the extracellular milieu of EGF ligands, small 6 kDa polypeptides made of about 50 residues, which eventually act in an autocrine or paracrine manner [87].

Many cytotoxic compounds have been covalently linked to EGF using the polypeptide as a vehicle for the delivery of these agents to a broad spectrum of cancer cells. Upon binding, EGF is internalized in the cell through receptor-mediated endocytosis, enabling the intracellular accumulation of the tethered cytotoxic compounds. Thus, EGF represents a natural place to start to target EGFR with photosensitizers (Table 1).

The bioconjugation of a photosensitizer to a protein is able to bypass many of the restrictions typical of photosensitizers, such as (i) the poor water solubility or low biocompatibility, (ii) the dependency of their ability to generate ROS on the features of the physiological environment, (iii) aggregation phenomena, (iv) the non-specificity of the cellular uptake, and (v) the poor pharmacokinetic or pharmacodynamic properties [42,88,89,90,91,92].

**Table 1:** EGFR-targeted PDT performed with EGF as the targeting agent.

Targeting Agent	PS	In Vitro Studies	In Vivo Studies	Ref
EGF	Disulfochloride aluminum phthalocy- anine [Pc(Al)], disulfochloride cobalt phthalocyanine [Pc(Co)]	MCF-7, B16 cells	Melanoma B16 cells in C57B1/6 mice	[93]

Bioconjugates of EGF with disulfochloride aluminum phthalocyanine [Pc(Al)] and disulfochloride cobalt phthalocyanine [Pc(Co)] were prepared, using EGF as a vector for targeted delivery of the phthalocyanines to cancer cells [93]. The molar ratio of the EGF-Pc(Al) and EGF-Pc(Co) bioconjugates was 1:1 [93]. It was demonstrated in vitro that both the bioconjugates showed phototoxic activity and that the phototoxicity of EGF-Pc bioconjugates exceeded the activity of the free Pc [93]. Intravenous injections of the EGF bioconjugate inhibited tumor development and increased the mean life span and mean survival time of experimental animals, while injections of free phthalocyanine had no effect on these parameters [93].

To improve the photosensitizer—EGF, ratio a support carrier able to bind multiple photosensitizers may be attached to the EGF. These conjugates should be constructed in a manner that allows EGF to retain its affinity for the EGF receptor as much as possible in order to maintain its innate targeting ability. (Table 2)

Therefore, EGF was conjugated with carrier molecules such as polyvinylalcohol (PVA), dextran (Dex) and human serum albumin (HSA) [94,95]. The phototoxicity of these systems, conjugated with Sn-(IV)chlorin e6 and targeted with EGF, were then compared to the conjugate of the photosensitizer and to PVA, Dex, and HSA alone [94,95]. The data demonstrated that the different carriers exert distinctive effects on (i) the affinity of EGF for its receptor and (ii) non-specific uptake [94,95]. In fact, it was observed that in the EGF-Dex-SnCe6 bioconjugate, EGF showed a dramatic decrease in affinity for the receptor in comparison with native EGF, while in the EGF-PVA-SnCe6 conjugate, EGF completely lost its affinity for the EGFR [94]. However, EGF-PVA-SnCe6 exhibited a higher photocytotoxicity than EGF-Dex-SnCe6, indicating that the carrier, more than EGF, plays a determinant role in the non-specific uptake of the photosensitizer by cells. Interestingly, in the case of the EGF-HSA-SnCe6 bioconjugate, only a moderate decrease in affinity for the EGF receptor was observed [95], resulting in potent EGF-dependent photocytotoxicity [95]. When the conjugate was incubated with an excess of EGF competitor, significant decreases in photocytotoxicity, cellular uptake, and ROS generation were observed, demonstrating that the photodynamic effect of EGF-HSA-SnCe6 relies on EGF-receptor-dependent intracellular accumulation [95].

EGF was also used as a targeting agent to decorate the surfaces of more complex nanoparticle assemblies. EGF was conjugated on the surfaces of chitosan nanoparticles, encapsulating curcumin^39^, or on the surfaces of gold nanoparticles functionalized with Ce6 [97,98]. EGF efficiently addressed the nanoparticles against EGFR-overexpressing cancer cells, showing superior phototoxicity when compared to free photosensitizers [96,97,98].

The major limitation to the use of EGF as a targeting agent is due to the reduction or even complete loss of its binding ability during the chemical modification process. In addition, the use of proteins for the targeted delivery of photosensitizers also has some other disadvantages, resulting from (i) their sensitivity towards pH, temperature, and organic solvents, which may lead to unfolding and loss of recognition properties during the bioconjugation procedures; (ii) high production costs; and (iii) sample heterogeneity, resulting in poor reproducibility.

### 3.2. EGFR-Targeting Peptides

Recently, the use of peptides as targeting elements has gained attention because these biomolecules (i) are easy to synthesize chemically at low cost; (ii) offer the possibility to conjugate drugs, tracers, radionuclides, and sensitizers in a chemically controlled manner [99]; (iii) are characterized by high binding affinity for the biological target; (iv) display low immunogenicity; and (v) have good pharmacokinetic and pharmacodynamic properties (i.e., enhanced penetration and diffusion into tissues and blood clearance), circumventing many of the drawbacks that proteins may exhibit.

Targeting peptides usually bind to surface-exposed tumor markers such as receptors, sugars, and components of the extracellular matrix. They can be identified through different approaches [100], including isolation of the receptor binding domain motif, molecular modeling, solid phase screenings of one-bead-one-compound (*OBOC*) peptide libraries [101], phage display [102,103], or even cell-free gene expression systems, such as ribosome display [104].

As a result, the conjugation of photosensitizers with EGFR-binding peptide ligands is an appealing method for boosting their biological efficacy. Several peptides have readily been used for PDT tumor-targeting approaches, including subcellular-targeted cancer therapy (STCT) strategies. Various peptides that selectively target EGFR have been isolated and successfully implemented in PDT (Table 3).

Using solid-phase synthesis, Ng et al., synthesized different bioconjugates by coupling Zn-phthalocyanine(ZnPc)-based photosensitizers with EGFR-targeting peptides, such as LARLLT (D4) [105,108], QRH* [110], and YHWYGYTPQNVI (GE11) [109]. The photophysical properties, cellular uptake, in vitro cytotoxicity, and in vivo biodistribution of the bioconjugates were examined. Peptide conjugation significantly improved the photodynamic efficacy and selectivity of the conjugated photosensitizer against cancer cells, with varying levels of receptor expression. Competitive assays showed a receptor-mediated internalization pathway of these bioconjugates, resulting in lysosome localization. It was shown that the intracellular localization of the adducts and the cell death pathways triggered by PDT may both vary according to the incubation period of the adducts. Photosensitization in the cell membrane caused the onset of necrotic events, likely due to membrane damage, while the photosensitization in the lysosomes appeared to trigger apoptosis. The peptide bioconjugates also demonstrated an enhanced tumor target selectivity in xenograft cancer models. Despite its small peptide sequence, QRH* gave the best performance.

To improve the proteolytic and metabolic stability, the bioactivity, and the binding specificity of the EGFR-targeting peptides, attempts to cyclize their peptide sequences were also made [111]. In fact, cyclic peptides are characterized by higher stability toward enzymatic proteolysis, while their rigid architecture improves the binding and recognition of the receptor. One of the conjugates containing a cyclic form of the epidermal growth factor receptor (EGFR)-binding peptide sequence CMYIEALDKYAC showed a higher photocytotoxicity than that of the analogues with a linear EGFR-targeting QRH* or GE11 [111]. The cyclic bioconjugate showed preferential uptake by two EGFR-positive cancer cell lines compared with two EGFR-negative counterparts, resulting in significantly higher photocytotoxicity [111]. Intravenous administration of this conjugate into tumor-bearing nude mice resulted in selective tumor localization and effective inhibition of tumor growth upon irradiation [111].

The conjugation of the photosensitizers to EGFR-binding peptide has been also implemented to create innovative responsive theranostic agents. Kim et al. [106,107] coupled a second-generation photosensitizer chlorin e4 (Ce4) with the EGFR-targeting peptide GE11 via a cleavable disulfide linker. The adduct resulted in a redox-responsive theranostic agent (RedoxT), which was usable for fluorescence imaging and photodynamic treatment of EGFR-overexpressing cells [106,107]. In particular, the amino acid tryptophan (Trp), present in the peptide, in the conjugated state quenches both the fluorescence emission and the ability to generate singlet oxygen of the Ce4 (OFF state). After the binding with EGFR, RedoxT is internalized and localizes in the lysosome, where the disulfide linker is cleaved by intracellular reducing agents (i.e., glutathione, GSH), triggering the release of Ce4s. Ce4, separated from the Trp-containing, GE11-targeting peptide, switches into an active form (ON state) and becomes highly fluorescent and phototoxic inside EGFR-overexpressing cells. A xenograft mouse model demonstrated the utility of RedoxT for in vivo NIR fluorescence imaging of EGFR-positive cancer cell lines.

EGFR-binding peptides can also be used to decorate and target bigger nanocarriers or adducts containing photosensitizers (Table 4). 

For example, the EGFR-binding peptide YHWYGYTPQNVI (GE11) was conjugated to the external carboxyl group of the bifunctional PEG layer of PEGylated gold nanoparticles (AuNPs; ca. 5 nm gold core). Silicon phthalocyanine Pc 4 was subsequently adsorbed onto the AuNP surface through N—Au bonding via the Pc 4 axial ligand’s terminal amine group [112]. When the photosensitizer is adsorbed on the AuNPs surface it is inactive; however, after its release, the photosensitization activity is recovered [112]. Generally, very few AuNPs are internalized by the targeted cells. However, targeting of Pc 4-loaded AuNPs to EGFR significantly improved (10-fold) the delivery of photosensitizers to brain tumors. This increase in photosensitizer delivery appeared to be mediated by a novel mechanism, in which the interaction of the GE11 targeting peptide with EFGR fostered a prolonged retention of AuNPs at the cell surface, allowing the hydrophobic Pc 4 moiety to be transferred to the cellular membrane [112]. In vivo studies showed that the GE11—AuNP—Pc 4 adduct crosses the BBB and BBTB efficiently, allowing transport of the photosensitizer selectively to tumors in the brain, reaching a maximum by 4 h post-injection, without accumulating in the healthy brain tissue [112]. Shorter peptides were also conjugated to AuNP—photosensitizer adducts to target EGFR-overexpressing cancer cells. These are particularly appealing due to their ease of synthesis, while maintaining good selectivity for the EGFR. Of these, AEYLR is of particular interest, as it shows a high binding affinity towards EGFR. The latter was conjugated to a 4 nm AuNP with a self-assembled mixed monolayer of the photosensitizer zinc phthalocyanine C11Pc and PEG [117]. The covalent binding of all components of this nanocarrier avoids any off-target phototoxicity due to desorption of the photosensitizer. Moreover, the EGFR-targeting peptide AEYLR was modified with a lysine residue at the C-terminus to allow the conjugation to the PEG (HS-PEG-COOH), and with a FITC-βAla at the N-terminus, allowing for imaging. This targeted nanosystem produces singlet oxygen upon irradiation at 633 nm, and a selective phototoxicity was observed for EGFR-overexpressing cells, displaying nanomolar potency and minimal dark toxicity [117].

Nanocarrier micelles are also able to entrap various photosensitizers and deliver them to tumors, especially when conjugated with EGFR target peptides, allowing for the targeting of EGFR-overexpressing tumor cells. For example, Pc 4-loaded poly(ethylene glycol)-poly(ɛ-caprolactone) (PEG-PCL) micelles were surface-modified with multiple copies of the GE11 peptide. When compared to non-targeted formulations, EGFR-targeted formulations resulted in greater intracellular absorption and subsequent PDT response (cell death) after photoirradiation in shorter time periods (10 min to 5 h) [113,114,115]. The formulation was further improved by tweaking variables such as the targeting ligand decoration density and the micelles’ Pc 4-loading extent, as well as the incubation dosage and photoirradiation parameters, in order to achieve maximal cell death in vitro [113,114,115]. Building on the promising in vitro data, the EGFR-targeted Pc 4 nanoformulation was also tested in vivo, where it resulted in significant intratumoral photosensitizer uptake and enhanced PDT response [113,114,115].

A similar strategy was employed using pH-responsive micelles, generated by the pH-responsive copolymer poly(ethylene glycol) methacrylate-co-2-(diisopropylamino)ethyl methacrylate (PEGMA−PDPA), used to entrap the photosensitizer Ce6 and functionalized with the EGFR-targeting peptide GE11 [116]. In the presence of Ce6/GE11^-(pH)^ micelles, Ce6 uptake by EGFR-overexpressing cells significantly increased due to GE11 specificity [116]. Moreover, Ce6 was released from Ce6/GE11^-(pH)^ micelles in tumor environments and lysosomes after EGFR-mediated endocytosis, leading to improved elimination of cancer cells in PDT [116]. In vivo experiments also confirmed that Ce6/GE11-^(pH)^ micelles specifically target and accumulate in EGFR-overexpressing tumors, following both passive and active uptake. This strategy allowed for both imaging and therapy, determining a significant suppression of tumor growth [116].

A very interesting approach for EGFR-targeted PDT was recently proposed by Eggleston and coworkers [118]. They developed an efficient delivery method for aminolevulinic acid (ALA), a prodrug for PDT, combining the potential of dendrimeric ALA ester derivatives for delivering a higher payload of ALA in a single prodrug entity adduct with a EGFR binding peptide to target selectively EGFR-overexpressing cells. In vitro results demonstrated the effectiveness of the designed peptide-targeted dendrimer structure, which was able to improve the release of ALA and to enhance the PDT activity in EGFR-overexpressing cells relative to the free prodrug [118].

The major limitation associated with the use of peptides as targeting agents is linked to their poor biochemical stability in vivo. In fact, they must be protected from enzymatic attack and proteolytic events by incorporation of unnatural or D-amino acids, cyclization, or other strategies to block the amino- and carboxyl-termini [99].

### 3.3. EGFR Small-Molecule Inhibitors

The conjugation of photosensitizers with small-molecule ligands specifically recognized by cancer cell receptors represents an alternative modality to enhance the selectivity and efficacy of PDT [119]. The use as a targeting moiety of a small-molecule ligand, characterized by a simple molecular structure and high stability in different solvents and experimental conditions, has many synthetic advantages when compared to biomolecules.

Many drugs that target and inhibit the receptor kinase activity of EGFR have been developed. Their chemistry and mode of action are beyond the scope of this work and have recently been excellently reviewed in the literature. Photosensitizers covalently conjugated to such small-molecule target-based anticancer drugs combine the phototoxicity of the photosensitizer with the excellent specificity of the small-molecule targeting agent (Table 5).

Erlotinib is a small-molecule EGFR tyrosine kinase inhibitor that specifically targets the ATP binding domain of the tyrosine kinase. A series of erlotinib-zinc(II)-phthalocyanine (ZnPc) conjugates were designed and synthesized [120,121,122]. Compared with free ZnPc, all the conjugates exhibited high specific affinity to EGFR-overexpressing cancer cells due to the presence of erlotinib, maintaining at the same time the high phototoxicity of the ZnPc core [120,121,122]. As a consequence, the developed conjugates displayed targeting photodynamic activities against EGFR-overexpressing cancer cells and specificity for tumors tissues in nude mice [120,121,122]. The choice of the correct linker proved critical to maintaining the maximum targeting capacity of erlotinib [120,121,122].

Analogously, to improve the selectivity of photosensitizer accumulation in EGFR-overexpressing cancer cells, chlorin e6 (Ce6) was conjugated with 4-arylaminoquinazolines, a class of molecules analogues to Vandetanib, a known tyrosine kinase inhibitor of both VEGFR-2 and EGFR [125]. The conjugate, compared to free Ce6, showed increased accumulation in cells with higher levels of EGFR expression, increased phototoxicity, and preferential accumulation in the tumor tissue of tumor-bearing mice [125].

The use of silicon phthalocyanine (SiPc) [123,124,126] as a photosensitizer has been shown to further improve the targeting strategies based on the exploitation of EGFR small-molecule inhibitors. In fact, the utilization of SiPcs enables the introduction of two small-molecular-target-based moieties at the axial positions, without chemical modification of the photosensitizer. Xue and coworkers synthesized and fully characterized silicon phthalocyanines di-substituted axially with erlotinib using a poly(ethylene glycol) (PEG) linker [123,124]. In vitro experiments showed that: (i) the PEG linker length has an important role on the photophysical and photochemical properties of the conjugate, affecting its phototoxicity in vitro; (ii) the erlotinib-based SiPcs have high cancer-targeting ability, due to the introduction of the two erlotinib moieties; (iii) the high phototoxicity of the SiPc core is maintained; (iv) the conjugates localize in lysosomes and mitochondria [123,124].

Very recently, the evidence that PDT targeting of specific organelles is able to trigger different cell death pathways and to influence phototherapeutic outcomes has received considerable interest. For example, mitochondrial and endoplasmic reticulum (ER)-localized photosensitizers cause selective photodamage to some proteins (i.e., m-TOR) involved in the apoptotic or autophagic process. SiPc offers the unique possibility to easily introduce two different small-molecular-targets, one for cell targeting and another to direct the subcellular localization. As the biggest organelle in eukaryotic cells, the ER is engaged in a variety of internal metabolic activities, including biosynthesis, sensing, and signal transmission, including protein folding and post-translation modification. Changes in ER function can cause a buildup of unfolded proteins, which can lead to ER stress. Excessive ER stress can induce tumor cells death, offering a novel and promising technique for cancer cell eradication. The ability of the methyl sulfonamide group to guide target molecules into the ER was recently shown. Xue and co-workers synthesized a multitarget photosensitizer that can selectively carry out photodynamic treatments in the ER of EGFR-overexpressing tumor cells [124]. They used the SiPc as a photosensitizer, while for the axial ligands on one side they used erlotinib and on the other side a methyl sulfonamide derivative [124]. Erlotinib improved the tumor-targeted specificity in cells overexpressing EGFR receptors. The attached methyl sulfonamide moiety directs the photosensitizer to the ER once it reaches the tumor site. In vitro experiments showed that the PDT irradiation triggered the production of cytotoxic ROS in the ER. This was shown to promote ER stress, upregulate intracellular Ca^2+^ ions levels, and decrease mitochondrial membrane potential, accelerating cancer cell death in a synergistic way [124].

In a different study, the same group used a similar approach to design a novel EGFR–mitochondria dual-targeted photosensitizer [126]. Again they used SiPc as the photosensitizer, conjugated this time with (i) gefitinib as target agent for EGFR-overexpressing cells and (ii) alkylated triphenylphosphonium cation (TPP^+^) for mitochondrial targeting [126]. As organelles particularly sensitive to photodamage, mitochondria represent some of the most effective sites of action to kill cells using PDT [129]. The double-targeted agent was mainly located in the mitochondria of the cancer cells, while SiPc and SiPc–gefitinib conjugate are preferably located in the lysosomes [126]. The phototoxicity of the double-targeted agent was considerably higher than the parent compounds and did not show remarkable cytotoxicity against normal cells, showing its excellent targeting ability against cancer cells and suggesting that mitochondrial localization achieved a more precise and effective photodynamic action than the non-targeted or single-targeted agents [126].

Additional functionalities can be also added to the PS-EGFR—ligand platform, for the development of multimodal imaging agents or combination therapy. Photosensitizers such as chlorines have fluorescence properties that are exploitable in imaging. Chlorine labelled with ^124^I represents an innovative agent characterized by multimodal imaging (positron emission tomography (PET) and fluorescence) and therapeutic potential (PDT) [127]. The in vitro and in vivo anticancer activity and imaging properties of iodinated photosensitizers with and without erlotinib were investigated in EGFR-positive cell lines and tumorous mice [127]. The erlotinib-conjugated iodinated chlorine showed EGFR target specificity that improved both the phototoxicity and imaging properties [127]. On the other hand, an efficient synergistic therapy was developed by synthesizing a compound (NBSNe) that combined a PS moiety, Nile blue with S-substitution (NBS), and an EGFR tyrosine kinase inhibitor (EGFR-TKIs), neratinib (Ne) [128]. In this case, neratinib was used not only to enhance the tumor targeting ability via conjugation with an EGFR -targeting group, but also to prevent the tumor metastasis during PDT, via chemotherapeutic action [128]. The NBSNe conjugate (i) was efficiently uptaken by cancer cells and generated ROS upon irradiation due to the presence of the NBS, resulting in robust phototoxicity [128], and (ii) significantly inhibited cell migration and invasion due to the action of the EGFR inhibitor (Ne) [128]. Interestingly, NBSNe inhibited tumor growth and suppressed cancer angiogenesis and metastasis in a tumor-bearing mouse model [128].

EGFR-targeting ligands can also be conveniently linked to the surfaces of PS-carrying nanoparticles (Table 6).

A chitosan derivative for targeting EGFR-overexpressing cells was created by chemically linking the Cy7 photosensitizer and erlotinib to chitosan, exploiting the reactive amine and hydroxyl groups that are naturally present on the chitosan backbone. Eventually, the polymeric chitosan derivative self-assembled to form theranostic nanoparticles [130]. Alternative formulations using the chitosan-erlotinib derivative as a targeting agent were also developed. In one of these formulations, the photosensitizer molecules are not chemically conjugated to chitosan but are instead encapsulated in the form of a nanoparticle (in this case indocyanine green (ICG) nanoparticles) by the chitosan–erlotinib derivative [131]. A more complex dual-responsive nanosystem was also obtained by loading mesoporous silica nanoparticles (MSN) with indocyanine green. Zinc oxide quantum dots were used to seal off the pores of the MSNs. The PS-loaded MSNs were then covered with the chitosan–erlotinib deriviatives cross-linked by disulfide bonds. The “gatekeeper” ZnO can be efficiently dissolved in the acidic environment of cancer cells, and the disulfide cross-linked polymer can be degraded in the reducing intracellular environment. Both events endow the release of the loaded indocyanine, creating a dual pH- and redox-responsive nanoparticle [132]. In all of these formulations, the nanoparticles specifically bind to the erlotinib-sensitive EGFR-overexpressing cells and release their cargo (erlotinib and photosensitizer) under specific conditions. The synergistic effect between the erlotinib-targeted therapy and photodynamic therapy resulted in the activation of the apoptotic pathway and cell cycle arrest [130]. Upon intravenous administration, the erlotinib-guided nanoparticles accumulated in EGFR-overexpressing cells, producing strong fluorescence, while upon NIR irradiation they significantly inhibited the growth of subcutaneously implanted EGFR-responsive tumors [130].

### 3.4. Anti-EGFR Antibodies

The use of monoclonal antibodies (mAbs), which first occurred more than 40 years ago [133], has represented a turning point in cancer therapy, leading to significant translational success, with dozens of mAbs now approved for clinical treatments. Most mAbs inhibit tumor cell proliferation or angiogenesis by binding selectively to surface receptors or ligands, ultimately interfering with the signal transduction pathways and promoting cell proliferation. Moreover, mAbs are also able to attract complement and cellular effectors of the immune system to the tumor. These responses are mediated by the unique features of the conserved Fc antibody domain. These mAbs are intensively investigated as targeting moieties for the vehiculation of cytotoxic drugs, theranostic compounds, and nanoparticles to the tumor microenvironment, with nine conjugates being approved by the US Food and Drug Administration (FDA) for the treatment of hematologic and solid tumors [134].

PSs are covalently linked to an antibody to provide tumor selectivity. When the PS is conjugated to an antibody targeting specific cell membrane receptors overexpressed in tumors, it accumulates selectively at doses able to induce phototoxic effects [135].

The first attempt to link a photosensitizer to a mAb targeting EGFR dates back to 1999, when mMAb 425, which recognizes an epitope localized on the extracellular receptor domain of EGFR, was used to conjugate Temoporfin (mTHPC) [136]. The in vitro results, as expected, showed increased phototoxicity of the mMAb 425-mTHPC adduct compared to free mTHPC due to the improved internalization efficiency of the photosensitizer.

Numerous combinations of photosensitizers and monoclonal antibody targeting EGFR were subsequently developed (i.e., chlorin e6 [137,138] and benzoporphyrin derivatives [139,140,141,142,143] conjugated to cetuximab), all demonstrating the ability to kill EGFR-overexpressing cells, without significantly affecting the EGFR-negative cells (Table 7).

A turning point was reached by the work of Kobayashi et al. [144], who developed a mAb-based photosensitizer activated by NIR light for targeted PDT. They used panitumumab, a mAb able to target EGFR, conjugated to IR700DX [144]. In vitro panitumumab—IT700DX conjugates (pan-IR700) led to rapid necrotic cell death of EGFR-overexpressing cells [144,145,146]. When co-cultures of receptor-positive and receptor-negative cells were treated, only the receptor-positive cells were killed, despite the presence of unbound mAb-IR700 in the culture medium [144,145,146]. In vivo, effective tumor shrinkage and prolonged survival was observed in mice treated with a single administration of mAb–IR700 compared to untreated control mice [144,145,146].

The complex mechanism of killing exerted by the panitumumab–IR700DX (pan-IR700) and cetuximab-IR700DX (cet-IR700) conjugates has been investigated in detail in recent years using a wide panel of in vitro and in vivo tumor models. It became immediately clear that the conjugation of a PS with mAb was not only a strategy able to improve the targeting capability of PDT, but also a means to potentiate anticancer antibody therapies (photoimmunotherapy—PIT) [171,172,173].

These studies set a series of hallmarks common to many mAb-PS adducts:

(1) Irradiation triggers rapid cell death, in turn causing membrane damage, which allows extracellular water to enter into cells, resulting in swelling, blebbing, and cell bursting [144,145,146];
(2) The mAb–PS conjugate generates singlet oxygen and reactive oxygen species, which elicit a rapid response in the cell [153];
(3) Alternative mechanisms of killing may take place. For example, immediately after light exposure, axial ligands of the IR700 molecule dissociate, promoting aggregation and leading to damage and rupture of the cellular membrane [162];
(4) A comparison between the performances of cetuximab and panitumumab as targeting agents for EGFR in PIT showed that in vitro cet-IR700 and pan-IR700 bind to EGFR-expressing cancer cells with nearly identical affinity levels, and both agents are capable of penetrating into 3D spheroids at the same rate [150]. These properties result in nearly identical PIT-induced phototoxicity in vitro [150]. In contrast, the two mAbs showed different pharmacokinetic effects, likely depending on their IgG subclasses—cetuximab is a chimeric IgG1 (13% mouse and 87% human), while panitumumab is a fully human IgG2 allotype;
(5) The tumor killing ability of the treatment is dependent on the dose and modality of light exposure, i.e., multiple NIR PIT cycles proved superior to a single treatment. In vivo, different modalities of light delivery were tested, from the use of interstitial light diffusers to implanted wireless LEDs [159,164,165,166];
(6) The PIT treatment causes a large increase (up to 24-fold compared with untreated tumors) in vascular permeability that facilitates the delivery of intravenous therapeutics, resulting in a synergy between PIT and chemotherapy. This phenomenon is referred to as super-enhanced permeability and retention (SUPR) [148,156,174];
(7) PIT treatment causes an “immunogenic cell death” (ICD) [160,171,172,173]. Upon PIT treatment, cancer-specific antigens and membrane damage markers are produced. These signals provoke the local activation of dendritic cells (DC), a type of antigen-presenting cell (APC) able to prime inactive or resting naïve T lymphocytes, leading to the commitment of the adaptive immune system and cell-mediated cancer cell killing. PIT could, therefore, have an advantage over conventional immunotherapies, which are hampered by heterogeneous or poor delivery of antibodies or immunoconjugates, since the cancer cells escaping the first line of irradiation-mediated ROS production could be cleared by the activated (cytotoxic) T cells. Moreover, since PIT can be repeatedly applied, multiple NIR–PIT treatments could also reinforce the APC-mediated priming of the cellular immune responses against the tumor [171,172,173];
(8) The therapeutic effects of NIR–PIT therapy can be monitored with several different imaging modalities. Exploiting the fluorescent properties of the used photosensitizers, it is possible to detect whether the antibody—PS conjugate has bound to the cancer cells and to set the proper light dosimetry, measuring the photobleaching of the PS. Fluorescence lifetime imaging and bioluminescence imaging can be used in pre-clinical studies, while ^8^F-fluorodeoxy glucose positron emission tomography (^18^F-FDG-PET) or MR imaging can assess early therapeutic changes after the treatment;
(9) PIT also holds great promise in assisting surgeons in the intraoperative and postoperative elimination of residual tumor patches following incomplete tumor resections [155]. As such, the conjugation of photosensitizers to mAbs might be suitable to improve the treatment and elimination of multiple tumor foci in larger areas;
(10) The preclinical validation of PIT was achieved in immune-deficient mice. Accordingly, the PIT-mediated triggering of the immune system was not accomplished until the first trials in humans, which resulted in better-than-expected results and was eventually repeated in immune-competent animal models. These results prompted new investigations, in which the PIT targeting of immunosuppressor cells within the tumor was explored. PIT resulted in further enhancement of the systemic and selective host immunity, leading to significant responses in distant metastases that were not irradiated by light. These results indicate that the combination of targeted PDT, with other immune-activating strategies, including PIT itself, provide systemic anticancer effects and long-term immune memory, skipping the adverse autoimmune effects often triggered by the use of immune checkpoint inhibitors.

Currently, the Cetuximab-IR700 conjugate is being tested in a phase 3 clinical trial on patients with recurrent head and neck cancers. PIT has also been fast-tracked by the US Food and Drug Administration (FDA). In parallel, the Japanese Pharmaceuticals and Medical Devices Agency recently approved the clinical use of an EGFR-targeted PIT adduct (ASP-1929; Akalux™, Rakten Medical Inc., San Diego, CA, USA) in association with a diode laser system. (BioBlade™, Rakten Medical Inc., San Diego, CA, USA)

Following in the footsteps of the success of mAb–PS conjugates, antibodies were also used as targeting agents for nanovectors and nanoparticles to improve site-specific delivery of high PS payloads. Anti-EGFR antibodies were conjugated to the surfaces of different kinds of nanoparticles, incorporating PSs such as polymeric micelles, nanoparticles, cerasomes, virosomes, hydrogels, and liposomes (Table 8) [175,176,177,178,179,180,181,182,183,184,185,186,187,188,189].

Even if improvements of the targeting or phototoxicity were sometimes observed, a role of the antibody in the improvement of the nanoparticle action was never clearly elucidated. In fact, the adsorption or the covalent linking of the antibody is a process that may have serious consequences for the recognition moiety and binding activity of the antibody. In addition, due to their large size, the conjugation of antibodies may strongly affect the distinguished chemical-physical features of the nanoparticles, such as their dimensions, surface charge, z-potential, stability, and biological identity in physiological environments. This may impinge on the binding and uptake of the nanovectors, irrespectively of the intrinsic recognition ability of the antibody.

From this point of view, smaller and biochemically more tractable targeting moieties (e.g., peptides, ligands, nanobodies, affibodies, and aptamers; see below) are probably a better choice for the targeting of nanoparticles. Only when a rational multivariant engineering approach was used to redirect a Cetuximab-targeted nanolipid adduct did the role of the antibody become evident [178].

In this latter case, the photoimmunonanocongiugates (PINs), built via careful modulation of antibody orientation and surface density grafting, demonstrated a high selectivity for cancer cells, with up to 100-fold preferential binding and up to 30-fold improvements in EGFR-specific photokilling of EGFR-overexpressing cells in 2D cellular cultures [178]. More importantly, the cetuximab–PINs demonstrated ~16-fold enhancement in molecular-targeted photodynamic destruction in heterotypic organoids [178].

Despite these recent successes, antibody therapies suffer from being primarily cytostatic and the need for prolonged administration with consequent side effects. Another major drawback of mAbs is their large size (150–160 kDa), which can further increase after conjugation with the nanoparticles. The larger size limits their penetration and diffusion into tumors [190]. Moreover, full-length antibodies require costly mammalian expression systems to maintain the correct glycosylation patterns [191]. Accordingly, smaller engineered antigen binding scaffolds such as the single-chain variable fragment (scFv; ~25 kDa) have been devised, which are also more amenable for production in prokaryotic expression systems, despite their lower affinities and stability [192].

Single-chain variable fragment (scFv) antibodies are engineered via the fusion of the heavy (V_H_) and light chains (V_L_) of immunoglobulins through a short polypeptide linker. Their use has become a standard technique, allowing compact but functional antigen-binding fragments in bacterial systems. As such, scFv antibodies play pivotal roles as therapeutic and diagnostic agents of human diseases, including cancer [193]. The limited size of scFv antibodies favors their penetration and diffusion compared to mAbs. However, they may be hampered by poor stability, lower solubility, and lower affinity, which could limit their clinical use [194]. Nevertheless, at least one scFv has been used in combination with various photosensitizers as EGFR-targeting moieties for PDT, attaining reliable results in vitro and ex vivo (Table 9).

A reference case for PDT is represented by the recombinant anti-EGFR antibody fragment scFv-425, which is able to bind to EGFR and be internalized upon binding by receptor-mediated endocytosis. Furthermore, scFv-425 has been conjugated to Chlorin e6 [195] (scFv-425-Ce6) and IR700DX [196,197,198] (scFv-425-IR700DX) and tested in EGFR-targeted PDT. The conjugates were characterized by excellent phototheranostic activity, with high phototoxicity against EGFR-expressing cells [195,196,197], allowing imaging of cancer cell lines [196,197,198] and human biopsies [196,197,198].

### 3.5. Anti-EGFR Nanobodies

Nanobodies (Nb) derive from immunoglobulin folds lacking the light chains normally present in conventional antibodies. They were serendipitously discovered in the *Camelidae* family, who produce them naturally in addition to conventional antibodies [199]. Consisting of a single engineered monomeric variable antibody (V_H_H) domain, they are also referred to as single-domain or heavy-chain-only antibodies (sdAb, HcAb). As with whole antibodies, they are able to bind selectively to specific antigens, although with a molecular weight of only 12–15 kDa, nanobodies are much smaller than common antibodies or even Fabs and single-chain variable (scFv) fragments (respectively ~50 kDa, and ~25 kDa in size). The smaller size is accompanied by a reduced interaction surface with the antigen-binding site (paratope). This results in the recognition of unique epitopes otherwise inaccessible to conventional antibodies, and sometimes in lower affinities [200]. Given their hydrophilic nature, small size, and increased stability over a wide range of chemical and physical conditions (temperature, pH, redox potential, presence of proteases, etc.), nanobodies are conveniently isolated via panning of phage display libraries and are easier to express in bacterial or yeast systems for bulk production [201]. This makes them ideal for nanobiotechnological purposes. The small size also accounts for their excellent tissue penetration properties, supporting their extensive application in cancer theranostics [202,203]. The downside for clinical use is their rapid clearance from circulation, due to their sizes being below the renal filtration cut-off. However, PEGylation [204] or conjugation to the Fc domain of conventional antibodies [205] has been shown to increase their retention. For nanobody conjugates and nanoparticles, the absence of the Fc domain may be advantageous, as it could decrease complement- and cell-mediated immune responses, which are responsible for rapid clearance of the nanoconjugates [206]. Because of the strong homology to the human V_H_3 gene family, nanobodies bear low immunogenic potential. Nevertheless, immunogenicity can become a problem, especially after repeated dosing. To overcome this translational hurdle, some nanobody scaffolds have been successfully “humanized” in their amino acid composition [207].

EGFR-targeting nanobodies have been isolated by phage display, also in competition with other specific ligands (i.e., EGF or cetuximab), leading to the identification of several excellent scaffolds that have been used to target EGFR-overexpressing cancers [208,209,210].

Nbs differ from mAbs in their use as targeting agents for anti-EGFR PDT (Table 10). On one hand there is the inability of the Nbs to trigger antibody-dependent cell cytotoxicity (photoimmunotherapy), while on the other hand EGFR-targeted Nbs demonstrated quicker accumulation in the tumor, a more homogenous distribution throughout the tumor, and a faster clearance of unbound molecules—ideal characteristics for delivering a photosensitizer. This aspect is particularly crucial for PDT treatment. In fact, compared to mAb-PS conjugates, the use of Nb-PS conjugates is expected to shorten the period between administration and light application (1–2 h, instead days), leading to more extensive tumor damage and reducing the risks of systemic side effects and long-term phototoxicity.

Monovalent (7D12) and biparatopic (7D12-9G8) Nbs targeting EGFR were conjugated to the theranostic agent IR700DX [211,212]. The EGFR-targeted NB-IR700DX conjugates retain the binding affinity and specificity of the Nbs, including after PS conjugation, and cell lines with varying expression levels of EGFR have been identified [211,212]. In low nanomolar concentrations, the NB-IR700DX conjugates cause cell death in EGFR-overexpressing cancer cells, whereas IR700DX alone or the Nb–PS conjugates in dark conditions does not induce toxicity [211,212]. Phototoxicity has been correlated to the level of EGFR expression in different cancer cell lines [211,212]. The use of the internalizing 7D12-9G8 biparatopic Nb resulted in increased phototoxicity, in fact the biparatopic Nb was able to induce receptor clustering and consequently faster endocytosis [211,212]. In vitro PDT assays with spheroids demonstrated the efficient cell killing ability of the Nb-conjugates, indicating that these low-molecular-weight constructs are characterized by an excellent ability to penetrate into the spheroid [213]. Ex vivo PDT assays with clinical ascites samples showed that the phototoxicity was restricted to the EGFR-positive subpopulation of cells, confirming the selectivity for anti-EGFR Nbs [213].

EGFR-targeted NB–IR700DX conjugates are selective and were able to induce selective cancer cell death in vivo in an orthotopic tumor model [212]. Both the EGFR-targeted NB–IR700DX conjugates quickly (1 h) and specifically accumulate in tumors, showing homogeneous distribution [212]. Upon the PDT treatment, the conjugates lead to pronounced tumor necrosis and to the infiltration of immune cells, with almost no toxicity in healthy tissues [212]. Interestingly, Nb–PS therapy leads to reduced fluctuation in the level of damage generated and an increase in tumor damage when compared to antibody—PS treatment. These findings were linked to the greater size of the antibodies, which hampered homogeneous distribution in vivo [212].

Permanent vascular effects, including vasoconstriction, reduced perfusion and leakage have also been observed in the tumor area after Nb-mediated PDT [215]. From a therapeutic standpoint, a therapy that includes both direct tumor cell killing and tumor vascular damage is likely to be the most successful. The combined use of a PS conjugated to an EGFR-targeted Nb and to nanobodies targeting vascular endothelial growth factor receptor 2 (VEGFR2), which is mainly overexpressed in the tumor vasculature, represents an alternative approach that may be able to potentiate the therapeutic response of the PDT treatment, damaging both the cancer cells and the tumor-associated vessels [217].

In addition, the first evidence of immunogenic cell death produced by Nb-PDT was recently published, indicating that antitumor immunity can be generated [216].

Nb-PDT treatment is involved in all three killing mechanisms on the basis of the PDT treatment: (i) generation of oxidative stress that can directly cause apoptosis and necrosis of cancer cells; (ii) destruction of the cancer-associated vasculature; (iii) activation of an acute inflammatory and induction of the host defense immune response. This can turn a local therapy that causes damages to the primary tumor into a systemic treatment that can combat metastases and prevent recurrences.

In view of the clinical translation, the Nb-based EGFR-targeted PDT in a 3D head and neck squamous cell carcinoma (HNSCC)-patient-derived model was also investigated [167]. For all organoids tested, the effect of nanobody-targeted PDT was more pronounced than that of antibody-targeted PDT [167]. The increased internalization of the Nb-PS is correlated with increased cellular damage [167]. A comparison between 7D12-IR700DX and 7D12-9G8-IR700DX conjugates indicated that the biparatopic nanobody 7D12-9G8- IR700DX was the most effective of the organoids tested [167]. The organoid response to EGFR-targeted PDT proved to be donor-dependent and tumor-specific, while induction of EGFR expression increased sensitivity to EGFR-targeted PDT [167]. The correlation between EGFR expression and response to EGFR-targeting PDT, as observed in 2D cell lines, was also confirmed in patient-derived organoids [167]. Since organoids express EGFR at comparable levels to primary patient tissue, these results are clinically relevant, as they suggest that EGFR levels could be a predictor for EGFR-targeting PDT [167]. Importantly, organoids grown from surrounding normal tissues showed lower EGFR expression levels than their tumor counterparts and were not affected by PDT. The theranostic performances of the NB-IR700DX conjugates could also be improved by conjugating both the photosensitizer IR700DX and the chelator DTPA for applications in nuclear imaging and photodynamic therapy. The binding, internalization, and light-induced toxicity of 7D12-IR700DX were retained, and in addition in vivo xenografts were visualized with both SPECT and fluorescence imaging [219].

Alternative PS were also conjugated to EGFR-targeted nanobodies, for example Ru^II^ polypyridyl complexes [214] or benzophenothiazine [220]. The last molecule is particularly interesting, because this type I photosensitizer can generate toxic superoxide or hydroxyl radicals under hypoxic conditions. This aspect is crucial, because tumor hypoxia significantly reduces the effectiveness of phototherapy due to the insufficient supply of oxygen to the tumor. In contrast, the 7D12-benzophenothiazine conjugates showed high specificity and toxicity towards EGFR-overexpressing cells both under normoxia and hypoxia [220]. The conjugate was further evaluated in vivo, showing tumor-targeting capacity and tumor suppression efficiency.

Finally, Nb-targeted PDT has also found application in oncological animal patients, aiming to treat spontaneous tumors with high biological relevance; in particular, cats with oral squamous cell carcinoma (OSCC). In this case the NB_A_ nanobody was used, conjugated with IR700DX [218].

Because of their tiny size, Nbs have a short circulation half-life, which might be troublesome for some applications. Attaching Nbs to the surfaces of nanoparticles increases the size of the conjugate, helping to solve the problem. This methodology allows for the simultaneous inclusion of many Nbs, as well as other targeting moieties and therapeutic components, into a single adduct, resulting in multimodal and multifunctional theranostic platforms (Table 11).

This approach was demonstrated using thermoresponsive diblock elastin-like peptides (ELP) that reversibly self-assemble into micellar structures to create well-defined 7D12-containing nanoparticles, with a size of 24 nm, which is small enough to extravasate and penetrate the intercellular spaces of tumors but big enough to escape quick clearance from the circulation. The 7D12-decorated ELP micelles fully retained their EGFR-binding capacity and were able to selectively target EGFR-overexpressing cancer cells. Upon incorporation of the photosensitizer IR700DX, the resultant nanoparticles caused EGFR-specific light-induced cell death.

7D12 was also conjugated on the surface of a ferritin (Ftn) nanocage, generating a novel targeted drug delivery system (7D12-Ftn). Photosensitizer molecules, i.e., manganese phthalocyanine (MnPc), were loaded into the ferritin cavity, and the MnPc@7D12-Ftn particles were efficiently internalized by EGFR-overexpressing cancer cells, but not by EGFR-negative cells. Upon laser irradiation, MnPc@7D12-Ftn selectively killed EGFR-positive cells by generating ROS, whereas it had minimal effect on the EGFR-negative cells.

Additionally, polymeric micelles based on benzyl-poly(ε-caprolactone)-b-poly(ethylene glycol) (PCLn-PEG; *n* = 9, 15, or 23) were used in PDT, encapsulating temoporfin (mTHPC) as a photosensitizer [39]. An EGFR-targeted nanobody (EGa1) was conjugated to the surfaces of the micelles [39]. An enhanced and specific uptake and an increased phototoxicity were observed for the mTHPC-loaded micelles decorated with the EGa1 nanobody as compared to non-targeted micelles on EGFR-overexpressing cancer cells [39].

Finally, a multifunctional nanoplatform able to improve the therapeutic efficacy of PDT on tumor hypoxia was achieved by encasing a photosensitizer (IR1048-MZ) and an enzyme (Catalase, Cat) into an mPEG-SS-PLGA polymer [223]. An anti-EGFR Nb was conjugated by disulfide linking to selectively target EGFR-overexpressing cells [223]. This construct proved able to efficiently destroy primary tumors in vivo upon PDT irradiation, inhibiting lung metastasis and prolonging mice survival [223].

### 3.6. Anti EGFR-Affibodies

An interesting alternative to antibodies or nanobodies is represented by affibodies. Affibodies are small (~6 KDa), engineered protein domains composed of 58 amino acids, folded in a stable a three-helix bundle. They are based on the scaffold of the protein A IgG-binding Z-domain of the bacterium *Staphylococcus aureus* [224]. This robust non-Ig scaffold harbors 13 randomizable residues that can generate libraries with an extremely large number of possible ligand variants (>10^20^ combinations), displaying variable surface-binding properties grafted on an identical backbone. In principle, they can be designed or screened to bind with high affinity to any given target [225].

Affibodies mimic antibodies and other immunoglobulin folds in function and binding affinity; however, they share with nanobodies some desirable advantages such as the smaller size and the better stability, simplifying production and purification from prokaryotic expression systems. As such, several affibody molecules have been extensively investigated as scaffolds for direct cancer imaging and treatment, including EGFR [226]. They can also redirect/vehiculate larger cargoes such as nanoparticles or liposomes for the selective targeting of cancer cells [227,228].

In the conjugation with the photosensitizer, the use of affibodies is particularly convenient because: (i) the robustness and refolding properties of affibodies make them amenable to conjugation conditions that denature most proteins (i.e., incubation at pH 11 at 60 °C for up to 60 min); (ii) they lack cysteine residues, while adding an additional cysteine to the molecule allow a precise site coupling. The high affinity (pM to nM range) of the affibody molecules to their targets, their small size (in vivo this results in fast clearance from the circulation with mostly renal excretion), and good tumor penetration make them ideal targeting agents to increase the concentration of the photosensitizers in the tumor site, while limiting toxicity in normal tissues. The photosensitizer IR700DX (IR700) was conjugated to the ZEGFR affibody (Ze), which has high specificity and affinity for EGFR to produce the Ze-IR700 conjugate [229] (Table 12).

Ze-IR700 conjugates bind to EGFR-overexpressing cells and are uptaken, localizing predominantly in lysosomes [229]. The in vitro phototoxicity of the Ze-IR700 conjugate was demonstrated, suggesting that PDT treatment predominantly induces lysosome-associated apoptosis [229]. In vivo, PDT exerted a Ze-IR700 dose-dependent tumor growth suppression.

PEGylated titania coated upconverting nanoparticles (TiO_2_-UCNs) were conjugated with anti-EGFR affibodies to specifically target EGFR-overexpressing cells (Table 13) [230].

When compared to unmodified TiO_2_-UCNs, the anti-EGFR-TiO_2_-UCNs were internalized more rapidly and efficiently (~3.8 folds) by EGFR-overexpressing cells.^123^ This was due to the presence of the affibodies, which trigger receptor-mediated endocytosis of these nanoparticles, causing instantaneous attachment of anti-EGFR-TiO_2_-UCNs to the EGFR receptor [230]. Selective killing of EGFR expressing cells was demonstrated in vitro. In another in vivo model, a significant delay in tumor growth and an improved survival rate were obtained using the anti-EGFR-TiO_2_-UCNs conjugate compared to conventional chlorin-e6 (Ce6) treatment [230].

A glutathione (GSH)-responsive nanogel was decorated with an anti-EGFR affibody for EGFR-targeted photodynamic therapy [231]. The pheophorbide A (PhA) photosensitizer was first conjugated onto a poly[(2-(pyridin-2-yldisulfanyl)ethyl acrylate)-*co*-[poly(ethylene glycol)]] (PDA-PEG) polymer through a disulfide bond [231]. The nanogel was then fabricated by crosslinking the PhA-conjugated polymer through disulfide bonds [231]. The aggregation of PhA in the nanogel resulted in fluorescence quenching and reduced ^1^O_2_ generation. The GSH-mediated activation of the PhA photoactivity made the nanogel very safe for in vivo applications, since the nanogel would remain in its inactive state in the bloodstream, where the GSH concentration is low (2–20 μM), and greatly reduce the phototoxicity to normal tissues [231]. After accumulating in tumor tissues and efficiently entering cancer cells through ligand–receptor interactions, the nanogel is rapidly and fully activated through the elevated GSH concentration (~10 mM), and both the fluorescence intensity and ^1^O_2_ generation capacity of PhA are reactivated [231]. Due to the overexpression of EGFR in the tumor, the anti-EGFR affibody-decorated nanogel showed high cellular uptake and PDT efficacy in EGFR-overexpressing cells. In vivo, the highest PDT efficacy and tumor growth inhibitory effects were observed with the affibody decorated PhA–nanogel as compared to non-targeted PhA–nanogel and free PhA.

### 3.7. Anti EGFR Aptamers

Aptamers consist of short single-stranded DNA (or RNA) sequences that receive considerable interest as targeting agents. Because they can fold in peculiar conformations and bind with high affinity to specific cell targets, aptamers have strong potential as biosensors and as theranostic tools in cancer treatment [232].

Selective aptamers are identified through SELEX (systematic evaluation of ligands by exponential enrichment) screening in vitro, consisting of a first binding step of a random aptamer library to the target, then washing and elution of the bound probes, followed by an amplification step [233]. Successive cycles of SELEX and counterselection allow the isolation of the high-affinity aptamers, which are eventually cloned and sequenced to identify the specific aptamer sequence. For RNA aptamer generation, an in vitro transcription step allows the generation of the RNA aptamers from a DNA library. After panning, the isolated RNA aptamers are reverse-transcribed into DNA and amplified by PCR in order to re-enter the SELEX cycle [234].

Various aptamers have been reported as binders of receptors involved in cell proliferation and tumorigenesis, including EGFR [235], and several have been shown to inhibit the ligand-dependent receptor activation [236].

Aptamers can be regarded as chemicals or synthetic “antibodies”, with distinct properties that offer them unrivalled benefits over antibodies: (i) aptamers may be selected in vitro for any given target, bypassing the restrictions of using cell lines or animals (aptamers may also be selected against toxic or non-immunogenic targets); (ii) aptamers are synthetic products, and as a consequence they can be produced and purified in large quantities (batch-to batch reproducibility); (iii) their synthesis in high chemical grade is simple; (iv) the low cost of production provided by modern oligonucleotide synthesizers

Aptamers are an excellent choice for the targeted delivery of photosensitizers because of their low immunogenicity, ease of chemical modification (they are very stable and may revert to their active conformation after denaturation), strong binding affinity to the target, and tiny size, which allows penetration in solid tumors [237].

The trimalonic acid-modified C_70_ fullerene (TF_70_) was conjugated with an aptamer (R13; Table 14) [238].

The R13 aptamer was obtained through screening against cancer cells overexpressing EGFR. The R13 aptamer maintained good binding ability, even after its conjugation with TF_70_ [238]. The PDT activity of the aptamer-guided fullerene photosensitizer (TF_70_-R13 conjugate) greatly improved as compared with TF_70_ [238]. The lysosomal location of the TF_70_-R13 conjugate enhanced the formation of intracellular ROS during irradiation, efficiently improving the killing activity.

An anti-EGFR DNA aptamer was conjugated to a fluorinated dendrimer (APF, Table 15) [239].

Due to the targeting ability of the aptamer and the strong oxygen-carrying capacity of the fluorinated dendrimer, APF could precisely bind to EGFR-positive cells and efficiently alleviate the tumor’s hypoxic microenvironment [239]. The dendrimer was used also as a drug carrier to encapsulate photosensitizers, i.e., hematoporphyrin (Hp), as well as drug molecules, i.e., gefitinib (Gef) [239]. Under laser irradiation, a significant increase in ROS production was observed intracellularly in EGFR-overexpressing cells. The synergistic anticancer effect was promoted by the concurrent application of PDT with the chemotherapeutic action of the gefitinib [239].

### 3.8. Refactored Anti-EGFR Phages

Bacteriophages (phages) are ubiquitous viruses that naturally infect bacteria. They are harmless to eukaryotic cells and have been widely used to treat antibiotic-resistant bacterial infections [240,241] or as phage display systems for the screening of protein and peptide interactions [242]. Icosahedric and filamentous phages such as Qβ, T4, T7, and M13/fd are receiving growing interest as nanobiotechnological platforms for theranostic applications, since they represent innovative, harmless, and effective biosensors and delivery vehicles [243,244].

The filamentous M13 bacteriophage in particular is an ideal carrier for a variety of agents, because of its small genome and the many genetic tools available. The possibility to genetically engineer all of the principal capsid proteins, including the minor coat pIII (5 copies at the tip of the virion) and the major coat pVIII (2700 copies covering the cylindrical capsid surface), make M13 a very convenient multifunctional and modular targeting platform. Single-chain and single-domain antibodies or other affinity ligands fused to pIII provide high affinity and cooperative binding to the target, while the multitude of functionalization sites on pVIII results in a very high loading capacity and multivalency. The possibility to conjugate this self-assembling bioscaffold with functional nanomaterials and photosensitizers represents a unique opportunity for tumor-targeting vectors [245]. When tumor-targeting moieties are genetically displayed or chemically conjugated on the capsid surface, the phage can be fostered to guide drugs or photosensitizers to cancer cells.

The use of phages has many advantages when compared to other targeting agents for PS: (i) phages represent flexible retargetable platforms; (ii) cost-effective phage production; (iii) targeting peptides identified in the literature by phage display can be directly used, as they are stabilized by the fusion to the phage proteins; (iv) multivalent display of targeting peptides and antibodies allows the binding affinity or avidity to be enhanced; (v) hundreds or thousands of PSs per binding event can be achieved as compared to only a few sensitizers for IgGs or single-chain antibodies, increasing sensitizer local concentrations in targeted cells; (vi) M13 efficiently translocates across the blood–brain barrier [246,247], which is an essential requirement if cerebral malignancies have to be targeted.

Ulfo et al. (Table 16) recently took a fully orthogonal approach to M13 to target EGFR-overexpressing tumor cells [248].

A short EGFR-targeting peptide (SYPIPDT) identified from the screening of a phage display peptide library [250] was genetically displayed in pentavalency at the phage tip, while the major pVIII capsomers were chemically conjugated with hundreds of Rose Bengal photosensitizers. This orthogonal nanoarchitectonical design kept the possible interference of conjugated sensitizers on the targeting moiety at minimal levels. Upon EGFR-mediated internalization, the engineered M13 derivatives were able to generate intracellular ROS species upon activation by ultra-low light intensities (2 mW/cm^2^). Notably, the killing activity on an EGFR-overexpressing A431 human epidermal carcinoma cell line was observed at picomolar concentrations of the phage vector.

The same EGFR-targeting vector was conjugated with chlorin e6 (Ce6) instead of Rose Bengal [249]. Upon laser irradiation (660 nm, irradiance 50 mW/cm^2^), this new phage platform was able to generate ROS via the type I mechanism and to kill SKOV3 and COV362 ovarian cancer cells, even at concentrations at which Ce6 alone was ineffective. As also reported for the phage—RB conjugates [248], further microscopic analysis showed an enhanced cellular uptake of phage—Ce6 conjugates compared to free Ce6 and their mitochondrial localization. Following irradiation, autophagy induction was detected in target cells, supporting the outstanding nanobiotechnological potential of M13 for receptor-targeted PDT of EGFR-positive ovarian cancer. 

## 4. Conclusions

The clinical relevance of EGFR-positive cancers is underscored by their global incidence. Immunotherapy with mAbs represents the gold standard for these tumors. However, there is a pressing need for novel therapeutic approaches because only a subset of patients respond to therapeutic mAbs. Moreover, many initially responsive cases become resistant within one year after having received the first treatment. Another problem with EGFR-targeting therapies, especially small-molecules inhibitors and some mAbs, is the induction of dermatologic side effects occurring in a significant fraction of treated individuals. EGFR-targeted PDT therapy may potentially exacerbate the side effects of the EGFR-inhibitor-targeting moiety.

However, unlike conventional tyrosine kinase inhibitors and mAb-based immunotherapies, EGFR-targeted PDT does not rely on a direct intrinsic inhibitor function on the EGFR. It only uses the EGFR as a selective dock to deliver PSs to the cancer cell, which allows the following actions: (i) offers the possibility of continuing to use target therapy against EGFR receptors, including for those cells that become resistant to immunotherapy or chemotherapy, because the development of resistance depends on a variety of downstream cellular mechanisms, while the structure of the receptors often remains unchanged; (ii) lower dosages of the EGFR-targeting molecule are needed, as selective docking needs to be achieved instead of functional inhibition of the receptor; (iii) the EGFR-targeted PDT treatment kills cancer cells through physical mechanisms that trigger autophagy, apoptosis, and membrane damage, thereby overcoming drug and immune resistance mechanisms that may afflict conventional treatments.

On the other hand, apoptotic or necrotic cell death induced by PDT may also release cell debris and neoepitopes (or self-antigens), leading to the production of autoantibodies. Inflammatory responses that may occur after PDT may affect self-antigen release, thereby stimulating autoimmune responses. In principle, these reactions should be prevented when considering repeated PDT treatments. However, it has been reported that the cancer risk is lower in patients with autoimmune diseases [251]. This suggests that autoantibodies against neoepitopes may counter cancer progression, tentatively also explaining the efficacy of PIT in distant, non-irradiated tumor metastases.

The double targeting strategy, which is receptor-targeted and laser-focused or -triggered, represents another advantage of minimizing the risk of side effects to healthy tissues as compared to immunotherapy or chemotherapy. Indeed, photoimmunotherapy has been shown to be accompanied by few and transient side effects, likely because of its highly targeted nature [171].

Nevertheless, the adverse effects associated with some EGFR-targeting moieties should be carefully considered to promote the safe use of PDT in clinical practice, especially for repetitive treatments. For example, the development of autoantibodies against the targeting moieties or against hapten–carrier adducts can induce an immune response. Chemical strategies such as PEGylation or dextranylation can be used to engineer the PS therapeutic carrier (i.e., protein, antibody, peptide) to be less immunogenic, “shielding” epitopes or altering antigen processing and presentation.

A different pitfall of PDT is the poor body penetration of light. However, many studies have shown that PDT can use longer wavelength light in the near-infrared (NIR) range, which penetrates tissues at greater depth, is less energetic, and is less harmful to other cells and tissues. In recent years, several NIR-light-excited PSs have been designed and synthesized to promote photosensitizing as well as photothermal processes, achieving improved tumor ablation via synergistic PTT–PDT effects [252,253]. Since the low levels of oxygen in cancer cells are one of the major limits for the effectiveness of PDT, synergistic PTT–PDT in hypoxic conditions can activate an alternative mechanism of cell killing, namely thermal ablation.

However, the few centimeters of light penetration in the body and the need for a light wave powerful enough to excite the sensitizers remain critical points for the application of PDT–PTT. Excitingly, increasing evidence indicates that the photosensitizers used in PDT can also be excited by the deeper penetrating ultrasound [254] in sonodynamic therapy (SDT).

Given the excellent results achieved, the knowledge gained for the design and synthesis of PS adducts selectively targeting EGFR may be readily transferred to the design of next-generation agents for EGFR-targeted PTT–SDT.

## Figures and Tables

**Table 2:** EGFR-targeted PDT performed with EGF as the targeting agent and conjugated with carrier molecules.

Targeting Agent	PS	Cargo	In Vitro Studies	Ref.
EGF	Sn(IV)chlorin e6 (SnCe6)	Dextran (Dex)	A431	[94]
EGF	Sn(IV)chlorin e6 (SnCe6)	Polyvinylalcohol (PVA)	A431	[94]
EGF	Sn(IV)chlorin e6 (SnCe6)	Dextran (Dex)	MDA-MB-468	[95]
EGF	Sn(IV)chlorin e6 (SnCe6)	Human Serum Albumin (HSA)	MDA-MB-468	[95]
EGF	Curcumin	Chitosan	MKN45, GES	[96]
EGF	Chlorin e6 (Ce6)	Gold nanoparticles	MDA-MB-468, MCF 10A	[97,98]

**Table 3:** EGFR-targeted PDT performed with anti-EGFR peptides.

Targeting Agent	PS	In Vitro Studies	In Vivo studies	Ref.
LARLLT	Zinc phthalocyanine (ZnPc)	A431, MCF-7	A431 cells in female BALB/c nude mice	[105]
YHWYGYTPQNVI	Chlorin e4 (Ce4)	PCA-SMCs, MDA-MB-231, MDA-MB-468, HCC70	MDA-MB-468 cells in mice	[106,107]
KLARLLT	Zinc phthalocyanine (ZnPc)	A431, A549, MCF-7, PC-3	A431 cells in BALB/c female nude mice	[108]
YHWYGYTPQNVI	Zinc phthalocyanine (ZnPc)	A431, MCF-7	A431 cells in BALB/c female nude mice	[109]
QRHKPREGGGGSK	Zinc phthalocyanine (ZnPc)	HT29, HEK-293, HepG2	HT29 cells in female Balb/c nude mice	[110]
Cyclic CMYIEALDKYAC	Zinc phthalocyanine (ZnPc)	HT29, HCT116, HeLa, HEK293	HT29 cells in female Balb/c nude mice	[111]

**Table 4:** EGFR-targeted PDT performed with anti-EGFR peptides conjugated with carrier molecules.

Targeting Agent	PS	Cargo	In Vitro studies	In Vivo studies	Ref.
YHWYGYTPQNVI	Silicon phthalocyanine Pc4	Gold nanoparticles	9L.E29 rat glioma cancer cells, engineered to overexpress EGFR	Human glioma (Gli36D5) cells in mice	[112]
CYHWYGYTPQNVI	Silicon phthalocyanine Pc4	PEG (poly(ethylene glycol))-PCL (poly(ɛ-caprolactone) micelles	A431, MCF-7, SCC-15,	SCC-15 cells in SCID mice	[113,114,115]
YHWYGYTPQNVIGGGGC	Chlorin e6 (Ce6)	Methoxypoly(ethylene glycol)/poly(ε-caprolactone) (mPEG–PCL)	HCT-116, SW620	CT-116 and SW620 cells in BALB/c athymic (nut/nut) mice	[116]
FITC-βAAEYLRK	Zinc phthalocyanine C11Pc	Gold nanoparticles	A549, HEK293		[117]
LARLLT	5-aminolevulinic acid (ALA) (prodrug of protoporphyrin IX)	Dendrimer	MDA-MB-231		[118]

**Table 5:** EGFR-targeted PDT performed with EGFR ligands as targeting agents.

Targeting Agent	PS	In Vitro Studies	In Vivo Studies	Ref.
Erlotinib	Zinc(II) phthalocyanine (ZnPc)	HepG2, HELF	A431 cells in nude mice	[120,121,122]
Erlotinib	Silicon phthalocyanine (SiPc)	HepG2, A549, PC-9, HELF		[123,124]
Vandetanib analogues (4- arylaminoquinazolines)	Chlorin e6 (Ce6)	A431, HeLa, CHO	CT-26 cells in Balb/c female mice	[125]
Gefitinib	Silicon phthalocyanine (SiPc)	A549, MDA-MB 468, HeLa, HELF		[126]
Erlotinib	Chlorin derivatives	UMUC3, T24	UMUC3 cells in SCID mice	[127]
Neratinib (Ne)	Nile blue with S-substitution (NBS)	MCF-7, 4T1, HCC827, H1650-M3, NIH 3T3	4T1 cells in Bal/bc mice	[128]

**Table 6:** EGFR-targeted PDT performed with EGFR ligands as targeting agents and conjugated with carrier molecules.

Targeting Agent	PS	Cargo	In Vitro Studies	In Vivo Studies	Ref.
Erlotinib	Heptamethine cyanine dye (Cy7)	Chitosan nanoparticles	A549, PC-9, H1975	A549, PC-9, or H1975 cells in female Balb/c-nude mice	[130]
Erlotinib	Indocyanine green (ICG)	Chitosan nanoparticles	PC9		[131]
Erlotinib	Indocyanine green (ICG)	Mesoporous silica nanoparticles (MSN)	A549, PC-9, and H1975	A549, PC-9, or H1975 cells in Balb/c nude mice	[132]

**Table 7:** EGFR-targeted PDT performed with anti-EGFR monoclonal antibodies.

Targeting Agent	PS	In Vitro Studies	In Vivo/Ex Vivo Studies	Ref.
mMAb 425	Temoporfin (mTHPC)	UM- SCC-11B, UM-SCC-22A, A431	HNX-OE in nude mice	[136]
Cetuximab	Chlorin e6 (Ce6)	A431, HCPC-1	Syrian Golden hamsters treated with 7,12-dimethylbenz(a)anthracene (DMBA)	[137]
Cetuximab	Benzoporphyrin derivative (BPD)	A431, 3T3-NR6		[139]
Cetuximab	Benzoporphyrin derivative (BPD)	A431, J774, 3T3-NR6, OVCAR-5		[140]
Panitumumab	IR700DX	A431	A431 cells in six-to-eight-week-old female homozygous athymic nude mice	[144]
Panitumumab	IR700DX	A431	A431 cells in six-to-eight-week-old female homozygous athymic nude mice	[145]
Panitumumab	IR700DX	MDA-MB-468luc	MDA-MB-468luc cells in six-to-eight-week-old female homozygote athymic nude mice	[146]
Panitumumab	IR700DX	A431	A431 cells in female nude mice	[147]
Cetuximab	Benzoporphyrin derivative monoacid ring A (BPD)	OVCAR-5, CHO-WT, CHO-EGFR		[141]
Panitumumab	IR700DX	A431	A431 cells in six-to-eight-week-old female homozygous athymic nude mice	[148]
Panitumumab	IR700DX	HER2 gene–transfected NIH/3T3; A431, Balb3T3/DsRed	A431 or Balb3T3/DsRed cells in six-to-eight-week-old female homozygote athymic nude mice	[149]
Cetuximab	IR700DX	A431, MDAMB468-luc	A431 and MDAMB468-luc cells in six- to eight-week- old female homozygote athymic nude mice	[150]
Panitumumab	IR700DX	A431, MDAMB468-luc	A431 and MDAMB468-luc cells in six- to eight-week- old female homozygote athymic nude mice	[150]
Panitumumab	IR700DX	A431, Balb3T3/DsRed	A431 and Balb3T3/DsRed in six-to-eight-week-old female homozygote athymic nude mice	[151]
Panitumumab	IR700DX	MDA-MB	MDA-MB-468luc cells in six-to-eight-week-old female homozygote athymic nude mice	[152]
Panitumumab	IR700DX	A431	A431 in mice	[153]
Cetuximab	IR700DX	MDAMB231, MDAMB468	MDAMB231 and MDAMB468 cells in six-to-eight-week-old female homozygote athymic nude mice	[154]
Panitumumab	IR700DX	SCC- 1-Luc	SCC- 1-Luc in athymic female nude mice, aged 5–6 weeks, tumor specimens obtained from histologically confirmed SCCHN patients	[155]
Panitumumab	IR700DX	A431	A431 cells in six-to-eight-week-old female homozygous athymic nude mice	[156]
Cetuximab	IR700DX	OE33, FLO-1, SW1573, MCF-7		[157]
Panitumumab	IR700DX	TCCSUP, 5637, RT4, T24, ScaBER, HT1197, HT1376, UMUC-3, SW780, A431, MDA-MB-453, RT112. Metastatic lines of T24, UMUC-3, T24T, FL3, SLT3, Lul-2. MGH-U3, UMUC-5, UOBL103, UPS 54	UMUC-5 and UMUC-3 cells in female Athymic Nu/Nu mice	[158]
Panitumumab	IR700DX		hEGFR TL transgenic mice	[159]
Cetuximab	IR700DX	A431		[160]
Cetuximab	IR700DX	Scc-U2, scc-U8, OSC19, A431		[161]
Cetuximab	IR700DX	Luciferase- and GFP-expressing A431, MDAMB468, 3T3/Her2, Calu3	A431-luc-GFP, 3T3/Her2-luc- GFP, MDAMB468-luc-GFP, or Calu3-luc-GFP cells in six-to-eight-week-old female homozygote athymic nude mice	[162]
Panitumumab	IR700DX	Luciferase- and GFP-expressing A431, MDAMB468, 3T3/Her2, Calu3	A431-luc-GFP, 3T3/Her2-luc- GFP, MDAMB468-luc-GFP, or Calu3-luc-GFP cells in six-to-eight-week-old female homozygote athymic nude mice	[162]
Panitumumab	IR700Dx	A431, H520	A431 and H520 cells in female athymic nude mice	[163]
Panitumumab	IR700DX	A431-luc	A431-luc in female homozygote athymic nude mice aged 6 to 8 weeks	[164]
Panitumumab	IR700DX	A431-luc	A431-luc in female homozygote athymic nude mice aged 6 to 8 weeks	[165]
Panitumumab	IR700DX	A431-GFP-luc	A431-GFP-luc cells in Balb/c Slc-nu/nu nude mice (six-week-old, females)	[166]
Cetuximab	IR700DX	A431, HeLa, HEK293T, UM-SCC-14C	Patient-Derived Head and Neck Cancer Organoids	[167]
Panitumumab	IR700DX	TCCSUP, 5637, RT4, T24, ScaBER, HT1197, HT1376, SW780, NIH/3T3, SK-BR-3, RT112. Metastatic lines of T24-T24T, FL3, SLT3. 253 J, UMUC-5, UMUC-1, MGH-U3.	SW780 in five-week old athymic Nu/nu female mice	[168]
Cetuximab	Benzoporphyrin derivative (BPD)	U25, U87		[142]
Cetuximab	IR700DX	OSC-19-luc2- cGFP	OSC-19 in female BALB/c athymic nude mice 12 weeks old	[169]
Cetuximab	Benzoporphyrin derivative (BPD)	OVCAR-5		[143]
Cetuximab	IR700DX	OSC-19-luc2-cGFP, scc-U2, scc-U8	OSC-19-luc2-cGFP in BALB/c nu/nu mice.	[170]
Cetuximab	Chlorin e6	L-929, Capan-1, Panc-1, Aspc-1	Capan-1 and Aspc-1 cells in 5-week-old BALB/c nude mice.	[138]

**Table 8:** EGFR-targeted PDT performed with anti-EGFR antibodies conjugated with carrier molecules.

Targeting Agent	PS	Cargo	In Vitro Studies	In Vivo Studies	Ref.
Anti-EGFR murine IgG2a antibody	Verteporfin	Poly [2-methacryloyloxyethyl phosphorylcholine-co-n-butyl methacrylate-co-p-nitrophenylcarbonyloxyethyl methacrylate] (PMBN) nanoparticles	A431, H69	A431, H69 cells in female BALB/cA nude mice	[175]
Anti-EGFR antibody (ab2430, Abcam Inc., USA)	Indocyanine green (ICG)	Ormosil PEBBLE nanoparticles		Female CD1 mice treated with 7,12-dimethylbenz(a)anthracene (DMBA)	[176]
Cetuximab	Pyropheophorbide-a derivative (PPa)	Micellar aggregate of Ac-sPPp (pyropheophorbide-a linked via a peptide to a short polyethylene glycol tail)	A431	A431cells in female athymic NCr-nu/nu mice, 4–5 weeks old,	[182]
Cetuximab	Temoporfin derivative (mTHPC)	ORMOSIL nanoparticles	HeLa, HeLa EGFR +, A431		[183]
αEGFR monoclonal antibody (MAB1095)	Chlorin e6 (Ce6)	Chimeric immunopotentiating reconstituted influenza virosomes (CIRIVs)	CAL-27	Syrian Golden hamsters treated with 7,12-dimethylbenz(a)anthracene (DMBA)	[185]
Cetuximab	IRDye800CW	Cerasomes	CT26-fLuc	CT26-fLuc in Male Balb/c mice	[186]
Cetuximab	Chlorin e6 (Ce6)	Methoxy poly(ethylene glycol)-b-poly(lactide) (mPEG-b-PLA) micelles	A431, HT-29		[187]
Anti-EGFR-monoclonal antibody (mAb) (cell signaling; Danvers, MA, USA)	Indocyanine green (ICG)	Perfluorocarbon double nanoemulsion	T24		[188]
EGFR antibody (EGFR (WB: 1:1000; MA5-13070, Thermo Fischer Scientific)	Chlorin e6 (Ce6)	Fucoidan and alginates with gellan gum hydrogel	HT-29		[180]
Cetuximab	Benzoporphyrin derivative monoacid A (BPD)	Pre-formed plain liposome (PPL)	Ovcar-5, CAMA-1, A431		[184]
Cetuximab	5,10,15,20-tetrakis(4-aminophenyl)porphyrin (TAPP)	Porphyrin-implanted carbon nanodots (PNDs)	HCC827, H23, MDB-MA-231, HBL-100, HeLa	MDA-MB-231 cells in nude mice	[189]
Cetuximab	Zinc Phthalocyanine, ZnPcOBP	Mesoporous silica nanoparticles	AsPC-1, PANC-1, MIA PaCa-2		[177]
VI Cetuximab	Benzoporphyrin derivative (BPD)	Nanoliposome (Nal)	A431, MIA PaCa-2 cells, OVCAR-5, T47D, CHO-WT, CHO-EGFR, PCAF	MIA Paca-2+PCAF in Swiss nude mice	[178]
Cetuximab	Benzoporphyrin derivative (BPD)	Nanoliposome (Nal)	A431, MIA PaCa-2, SCC-9, T47D, CHO-WT, SKOV-3		[179]
Cetuximab	Benzoporphyrin derivative (BPD)	Nanoliposome (Nal)	OVCAR-5, U87, J774		[181]

**Table 9:** EGFR-targeted PDT performed with anti-EGFR scFV.

Targeting Agent	PS	In Vitro Studies	In Vivo/Ex Vivo Studies	Ref.
scFv-425	Chlorin e6 (Ce6)	A431, MDA- MB468, MDA-MB-231, SiHa, CHO-K1		[195]
scFv-425	IR700DX	MDA-MB-468, MDA-MB-453, MDA-MB-231, Hs758T, MCF-7	Human breast cancer biopsies and normal breast tissues	[196]
scFv-425	IR700DX	A431, HEK-293T, A2058		[197]
scFv-425	IR700DX	OVCAR-3, SKOV-3, IGROV-1, A2780	Human ovarian cancer biopsies and ascite samples	[198]

**Table 10:** EGFR-targeted PDT performed with anti-EGFR nanobodies.

Targeting Agent	PS	In Vitro Studies	In Vivo Studies	Ref.
7D12, 7D12-9G8	IRDye700DX	3T3 2.2, 14C, A431, HeLa		[211]
7D12, 7D12-9G8	IRDye700DX	OSC- 19-luc2-cGFP, HeLa, SW620	OSC-19-luc2-cGFP cells in nude Balb/c female mice	[212]
7D12	IRDye700DX	A431, E98, SKOV-3	Clinical ascites samples	[213]
7D12, 7D12-9G8	IRDye700DX	A431, HeLa, HEK293T, UM-SCC-14C	Patient-Derived Head and Neck Cancer Organoids	[167]
7C12	Ru^II^ Polypyridyl	A431, MDA-MB 435S		[214]
7D12, 7D12-9G8	RDye700DX	OSC-19-luc2-cGFP	OSC-19-luc2-cGFP cells in female BALB/c nude mice	[215]
7D12, 7D12-9G8	IRDye700DX	A431, scc-U8		[216]
7D12	IRDye700DX	MS1, OSC		[217]
NBA	IRDye700DX	SCCF1, SCCF2, SCCF3, HeLa, MCF7		[218]
7D12	IRDye700DX	A431	Mice bearing A431 xenografts.	[219]
7D12	Benzophenothiazine	A431, 4T1, MCF-7, HeLa	4T1cells in female Balb/c mice	[220]

**Table 11:** EGFR-targeted PDT performed with anti-EGFR nanobodies conjugated with carrier molecules.

Targeting Agent	PS	Cargo	In Vitro Studies	In Vivo Studies	Ref.
7D12	IR700DX	Elastin-like peptides (ELP) diblock polypeptide nanoparticles	A431, E98		[221]
7D12	Manganese phthalocyanine (MnPc)	Ferritin	A431, MCF-7		[222]
EGa1	Temoporfin (mTHPC)	benzyl-poly(ε-caprolactone)-b-poly(ethylene glycol) (PCLn-PEG) micelles	A431, HeLa	A431 cells in female Balb/c nude mice,	[39]
7D12	IR1048-MZ	mPEG-SS-PLGA-SH Nanoparticles	A549	A549 cells in female BALB/c mice	[223]

**Table 12:** EGFR-targeted PDT performed with anti-EGFR affibody.

Targeting Agent	PS	In Vitro Studies	In Vivo Studies	Ref
Anti EGFR-specific affibody (Z_EGFR:1907_)	IR700DX	COLO205, COLO 320 DR, COLO 320 HSR, LS174T, HT29, HCT-8, LOVO, RKO, LS180, T84, HCT116	COLO 205, LS174T, HT29 cells in 4- to 6-week-old female BALB/c nude mice.	[229]

**Table 13:** EGFR-targeted PDT performed with anti-EGFR affibodies conjugated with carrier molecules.

Targeting Agent	PS	Cargo	In Vitro Studies	In Vivo Studies	Ref.
Anti-EGFR Affibody	TiO_2_	Core–shell nanoparticle—titanium dioxide (TiO_2_) on a NaYF4:Yb, Tm UCN core	OSCC, A431, MCF-7,	OSCC cells in 6–8 week female Balb/c nude mice	[230]
Anti-EGFR Affibody	Pheophorbide A (PhA)	Poly[(2-(pyridin-2-yldisulfanyl)ethyl acrylate)-co-[poly(ethylene glycol)]] (PDA-PEG) nanogel	UMSCC 22A	UMSCC 22A cells in female Balb/c nude mice (8–10 week old)	[231]

**Table 14:** EGFR-targeted PDT performed with an anti-EGFR aptamer.

Targeting Agent	PS	In Vitro Studies	In Vivo Studies	Ref.
anti-EGFR DNA R13 aptamer 5′-TTT ATG GGT GGG TGG GGG GTT TTT; S14, 5′-GAT TGT CCC CGC GCC TGG TTG AAG	Trimalonic acid-modified C_70_ fullerene (TF_70_)	A549		[238]

**Table 15:** EGFR-targeted PDT performed with an anti-EGFR aptamer conjugated with carrier molecules.

Targeting Agent	PS	Cargo	In Vitro Studies	In Vivo Studies	Ref.
Anti-EGFR DNA aptamer 5′ -COOH- TGA ATG TTG TTT CTC TTT TCT ATA GTA-3′ (Apt)	Hematoporphyrin (Hp)	Fluorinated dendrimer	Helf, NSCLC PC-9, H1975		[239]

**Table 16:** EGFR-targeted PDT performed with phages expressing anti-EGFR peptides.

Targeting Agent	PS	Cargo	In Vitro Studies	In Vivo Studies	Ref.
SYPIPDT peptide in fusion with p3 phage protein	Rose Bengal	M13 phage	A431		[248]
SYPIPDT peptide in fusion with p3 phage protein	Chlorin e6 (Ce6)	M13 phage	SKOV3, COV362		[249]
